# Effects of Acute Caffeine Supplementation on Physical, Sport-Specific, Physiological, Perceptual, and Cognitive Outcomes in Female Team-Sport Athletes: A Three-Level Meta-Analysis

**DOI:** 10.3390/nu18152429

**Published:** 2026-07-24

**Authors:** Hai Li, Ming Chen, Mingnan Zhuang, Hengzhi Deng, Haiying Wang, Hansen Li

**Affiliations:** 1 School of Sport Training, Henan Sport University, Zhengzhou 450044, China; 18530010702@163.com; 2 School of Physical Education, Kunming University of Science and Technology, Kunming 650500, China; chenming061010@gmail.com; 3 College of Sports Training, Tianjin University of Sport, Tianjin 301617, China; iammnz@163.com; 4 Department of Sports and Exercise Science, Hebei Institute of Physical Education, Shijiazhuang 050041, China; 5 Faculty of Sports and Exercise Science, University of Malaya, Kuala Lumpur 50603, Malaysia; denghengzhi66@gmail.com; 6 School of Physical Education, Sichuan Agricultural University, Ya’an 625014, China; hanson-swu@foxmail.com

**Keywords:** caffeine, ergogenic aid, sports nutrition, perceived exertion, crossover trial, female athlete health, team-sport athletes, three-level meta-analysis

## Abstract

Background and Objectives: Evidence on the acute effects of caffeine in female team-sport athletes is limited. This study aimed to quantify the acute effects of caffeine on female team-sport athletes across physical performance, sport-specific performance, physiological responses, perceptual responses, and cognitive performance and determine moderators. Methods: PubMed and Web of Science were systematically searched for randomized, placebo-controlled crossover trials of acute, dose-defined caffeine in female team-sport athletes. Effect sizes were expressed as Hedges’ g and synthesized within each domain using a three-level CHE model with CR2 cluster-robust variance estimation and Satterthwaite degrees of freedom; 95% confidence intervals and prediction intervals were reported. Risk of bias (RoB 2), methodological quality (PEDro), and certainty of evidence (GRADE) were assessed. Results: Twenty-six studies were included, comprising 26 randomized, blind, placebo-controlled crossover trials. As a descriptive cross-domain summary, acute caffeine intake showed a small overall effect when all available domains were pooled (Hedges’ g = 0.24, 95% CI 0.13 to 0.35); however, because these domains represent different constructs, domain-specific estimates were considered the primary interpretable findings. For physical performance, caffeine showed a small favorable effect (g = 0.32, 95% CI 0.22 to 0.42), with statistical evidence for repeated sprint ability (g = 0.43), agility or change in direction (g = 0.42), sprint or speed performance (g = 0.39), anaerobic power (g = 0.30), and jumping performance (g = 0.29). For sport-specific performance, the pooled estimate indicated a small favorable effect but did not reach conventional statistical significance (g = 0.36, 95% CI: −0.01 to 0.73). Exploratory sub-indicator analyses, each based on only a few studies, suggested possible favorable directions for sport-specific locomotion or running performance (g = 0.46) and throwing or ball-speed outcomes (g = 0.19), whereas evidence was insufficient for shooting or scoring accuracy. Physiological outcomes were direction-aligned to the value conventionally regarded as favourable for each indicator (i.e., lower heart rate, lactate, and glucose), so that positive values denote the conventionally favourable direction; because the domain aggregates indicators of differing clinical desirability, its pooled value is a descriptive summary only and showed no clear domain-level direction (g = −0.05, 95% CI −0.31 to 0.21). The heart rate submetric showed a statistically detectable difference (g = −0.30), indicating that caffeine increased heart rate, the expected pharmacological response to a stimulant rather than an adverse effect; there were no clear effects on blood lactate or blood glucose. Perceptual responses improved modestly (g = 0.42), largely through reduced rating of perceived exertion (g = 0.35). Cognitive performance, based on only four studies with low degrees of freedom and wide uncertainty, showed a nonsignificant direction (g = 0.37) with no clear evidence for reaction time or cognitive accuracy, and these data should not be interpreted as showing that caffeine improves cognitive performance in female team-sport athletes. Exploratory moderator analyses did not provide statistically reliable evidence of moderation by caffeine dose, timing of intake, formulation or source, sport type, competitive level, habitual caffeine intake, or blinding status. Conclusions: Available evidence most consistently supports small, outcome-specific benefits of acute caffeine for physical performance and reduced perceived exertion. Evidence for sport-specific skills and cognitive outcomes remained uncertain, based on few studies per outcome and not supporting a general benefit. Current data do not support dose-, timing-, formulation-, habitual-intake-, sport-, or population-stratified recommendations. Future trials should be adequately powered, prospectively report menstrual-cycle phase, hormonal-contraceptive use, habitual caffeine intake, and adverse symptoms, and verify the integrity of blinding.

## 1. Introduction

Team-sport performance is not determined by a single physical capacity, but by the repeated integration of locomotor, neuromuscular, technical, and perceptual demands during competition. Time-motion and tracking evidence shows that soccer, basketball, handball, field hockey, futsal, and volleyball involve substantial running volume, sprint exposure, high intensity running, accelerations, decelerations, activity changes, cutting, lateral movement, and jumping, with demands varying by sport, competitive level, sex, and playing role [1,2,3]. Repeated sprint performance is also relevant because short sprints separated by incomplete recovery are common in team sports, and the ability to reproduce sprint output depends on metabolic factors, such as phosphocreatine recovery, oxidative capacity, and hydrogen ion buffering, as well as neural factors, including muscle activation and recruitment strategy [4,5]. These physical demands occur alongside technical execution and tactical decision making, and are often performed under accumulated fatigue, opponent pressure, and limited recovery opportunities. Competition structure also creates periods in which hydration, carbohydrate availability, muscle temperature, perceived effort, and cognitive readiness may influence subsequent performance, especially during later match phases and around halftime [6,7]. Therefore, acute nutrition and supplementation strategies in team ball sports should be evaluated according to whether they address plausible constraints on match performance, rather than only by their effects on isolated fitness tests [8,9].

Within this framework, caffeine (1,3,7-trimethylxanthine) is a relevant candidate because its proposed actions, including adenosine receptor antagonism, reduced perception of effort, increased alertness, attenuated pain perception, and possible effects on neuromuscular function, correspond to several limiting factors in intermittent team-sport [10]. Caffeine is commonly studied at doses of approximately 3 to 6 mg per kg body mass, with intake often timed around 60 min before exercise, although the optimal timing depends partly on the delivery form [10]. Evidence in team sports suggests that acute caffeine intake can improve some match-related physical outputs, including sprint actions, high intensity running, accelerations, decelerations, body impacts, and selected offensive actions, whereas effects on technical performance are less consistent because passing, shooting, ball control, and tactical decisions are more dependent on sport specificity, ecological validity, opponent interaction, and athlete skill level [11,12]. These outcomes can be grouped into three conceptually distinct categories: physical performance (e.g., jumping, sprinting, and repeated high-intensity actions), sport-specific technical and tactical skills (e.g., passing, shooting, ball control, and decision-making), and cognitive performance (e.g., reaction time, attention, and processing accuracy). Caffeine’s ergogenic effects are generally more consistent for physical outcomes than for technical skills which depend heavily on sport specificity, opponent interaction, and skill level whereas its effects on sport-relevant cognitive outcomes remain far less established.

Although caffeine has a substantial evidence base in exercise and sport performance, its applicability to female team-sport athletes remains insufficiently defined. This limitation is primarily methodological rather than speculative. In an audit of evidence-based performance supplement studies including 1826 studies and 34,889 participants, women accounted for only 23% of all participants, only 34% of studies included at least one female participant, 0% to 8% used female-only samples across supplement categories, and only 0% to 2% were designed to compare responses between sexes [13]. Consequently, much of the current supplement evidence, including evidence on caffeine, is still derived from male or mixed-sex samples, limiting direct inference for female athletes.

A female-specific synthesis is therefore warranted not because women necessarily respond to caffeine through a fundamentally different physiological mechanism, but because the precision, boundary conditions, and contextual modifiers of the available evidence remain uncertain. In female athletes, menstrual-cycle characteristics, hormonal-contraceptive use, habitual caffeine intake, symptom burden, sleep, and recovery kinetics may influence performance testing, perceived exertion, fatigue, recovery, and tolerance of supplementation. Although average menstrual-cycle phase differences in exercise performance may be small, current studies remain limited by methodological heterogeneity and inconsistent verification of cycle phase; therefore, these variables remain relevant for study design, interpretation, and translation [14]. In female team-sport athletes, caffeine-related outcomes extend beyond isolated strength or endurance measures to include physical, sport-specific, perceptual, and cognitive indicators expressed within sport-specific tasks and under intermittent fatigue, and trials in female samples have reported possible benefits for several of these outcomes [13,15,16].

Existing reviews have begun to synthesize evidence on caffeine in female team-sport athletes, but several questions remain unresolved. Gomez-Bruton et al. included 18 randomized crossover trials and reported benefits for selected sport-specific, neuromuscular, and match-related outcomes, but no clear effects for several agility, repeated-sprint, or perceptual outcomes [15]. A more recent review in female basketball players included seven studies using caffeine doses from 2.1 to 9 mg·kg^−1^ and reported possible benefits for power output, but inconsistent or unclear effects on shooting accuracy, dribbling sprint performance, jump height, agility, sprint performance, anaerobic capacity, perceived fatigue, and physiological or biochemical outcomes [16]. Together, these findings suggest that caffeine effects in female team sports are likely outcome- and context-dependent rather than uniformly beneficial or null. However, previous syntheses remain limited by the small number of female-only trials, uneven coverage across sports and outcome domains, and inconsistent reporting of key moderators, including dose, timing of intake, training status, habitual caffeine intake, menstrual-cycle phase, and hormonal-contraceptive use. In addition, conventional two-level random-effects models may not adequately account for statistical dependence among multiple effects extracted from the same study, such as when the same participants complete several physical, sport-specific, perceptual, and physiological tests within one trial [17,18]. Treating these correlated effects as independent can underestimate standard errors and overstate precision. As evidence in female team- and intermittent-sport settings has expanded, an updated synthesis is warranted to integrate a broader set of outcomes, model within-study dependence more appropriately, and clarify where caffeine effects are most consistent, where they remain uncertain, and which study or athlete characteristics may modify the estimated effects.

Accordingly, this systematic review and meta-analysis aimed to evaluate the acute effects of caffeine ingestion on physical performance, sport-specific skills, physiological responses, perceptual responses, and cognitive performance in female team-sport athletes. Where data permitted, subgroup and sensitivity analyses examined whether sport, caffeine dose, timing of intake, training status, habitual caffeine intake, menstrual-cycle phase, and hormonal-contraceptive use influenced the estimated effects.

## 2. Methods

### 2.1. Research Protocol and Registration

The design, conduct, and reporting of this systematic review and meta-analysis followed the Preferred Reporting Items for Systematic Reviews and Meta-Analyses (PRISMA 2020) statement in Supplementary Table S1 [19]. The protocol was prospectively registered on the Open Science Framework on 21 May 2026 (OSF; https://osf.io/6hqfp, 10.17605/OSF.IO/QZYWT). The review was conducted in accordance with the prospectively registered protocol, with one post-registration refinement to the review title and scope. The registered protocol, entitled “Acute and Chronic Effects, and Safety of Caffeine Supplementation in Adult Female Team-Sport Athletes: An Updated Systematic Review and Meta-Analysis with Menstrual-Cycle and Hormonal-Contraceptive Moderators,” included both acute and chronic caffeine supplementation. The final review was retitled “Effects of Acute Caffeine Supplementation on Physical, Sport-Specific, Physiological, Perceptual, and Cognitive Outcomes in Female Team-Sport Athletes: A Three-Level Meta-Analysis” and restricted to acute caffeine supplementation. This refinement was introduced to maintain a clinically and methodologically coherent synthesis of acute effects and to permit outcome-domain-specific three-level meta-analysis. Apart from this explicitly reported scope refinement, the final search strategy, outcome grouping, statistical model, and subgroup and sensitivity analyses were consistent with the registered protocol.

### 2.2. Search Strategy

Before the formal search, one reviewer conducted a pilot search to calibrate the search terms and conceptual boundaries. Two reviewers then independently and systematically searched PubMed/MEDLINE and Web of Science from database inception to 30 April 2026. These two databases were selected because PubMed/MEDLINE provides broad coverage of biomedical, nutrition, exercise-supplementation, and sports-medicine research, whereas Web of Science provides multidisciplinary coverage and citation indexing across sport science, exercise physiology, and nutrition journals. To improve retrieval beyond the two electronic databases, the reference lists of included studies and relevant reviews were also hand-searched by backward citation tracking. Nevertheless, we acknowledge that this database coverage was not exhaustive and that eligible records indexed only in databases such as SPORTDiscus, Scopus, Embase, the Cochrane Library, trial registries, or other regional databases may have been missed.

The search strategy was constructed from four concept blocks combined with Boolean logic: (i) population and sex (female[MeSH], women, woman, female*, girl*, etc.); (ii) sport and participants (athlete*, player*, “team-sport*”, basketball, soccer, football, volleyball, handball, rugby, hockey, futsal, netball, lacrosse, “water polo”, softball, etc.); (iii) intervention (caffeine[MeSH], caffeine, caffeinated, “1,3,7-trimethylxanthine”, “caffeine ingestion”, “caffeine supplementation”, “caffeine gum”, coffee, “energy drink*”, etc.); and (iv) study design (random*, placebo, crossover, “cross-over”, trial, intervention, “double blind”, “single blind”, “placebo-controlled”, etc.). The full database-specific search strings, including exact syntax, search dates, filters, and limits, are provided in Supplementary Table S12.

No publication-year restriction was applied. No language restriction was applied at the search stage; however, because accurate extraction of methodological details and numerical outcome data was required, only studies with an available English full text were eligible at the screening stage. Records excluded because the full text was unavailable and records excluded solely because an English full text was unavailable were considered separately in the PRISMA flow diagram. Conference abstracts were not included as eligible reports but were used only, when relevant, to trace a corresponding full-text article.

### 2.3. Eligibility Criteria (PICOS)

Inclusion and exclusion criteria were specified a priori using the PICOS framework (Table 1). “Team ball sports” were operationally defined as ball sports characterized by team competition and intermittent high-intensity exercise (basketball, volleyball, soccer, handball, rugby, softball, water polo, and similar). “Acute supplementation” referred to immediate testing after a single dose, to distinguish it from chronic protocols involving repeated dosing over several days. When a single study recruited female athletes from more than one such sport within the same sample, its sport was coded as “mixed team sports” a sample-composition label denoting a heterogeneous team-sport sample rather than a distinct sport or a mixed-sex sample, and it was not treated as a separate sport category in the synthesis. Regarding sex, only outcome data derived exclusively from female athletes were used: studies conducted entirely in female samples were included directly, whereas for mixed-sex studies only the female-specific results were used, and only when the female subgroup’s means, standard deviations, and sample sizes were reported separately or could be reconstructed without ambiguity.

### 2.4. Study Selection

**Figure 1:**
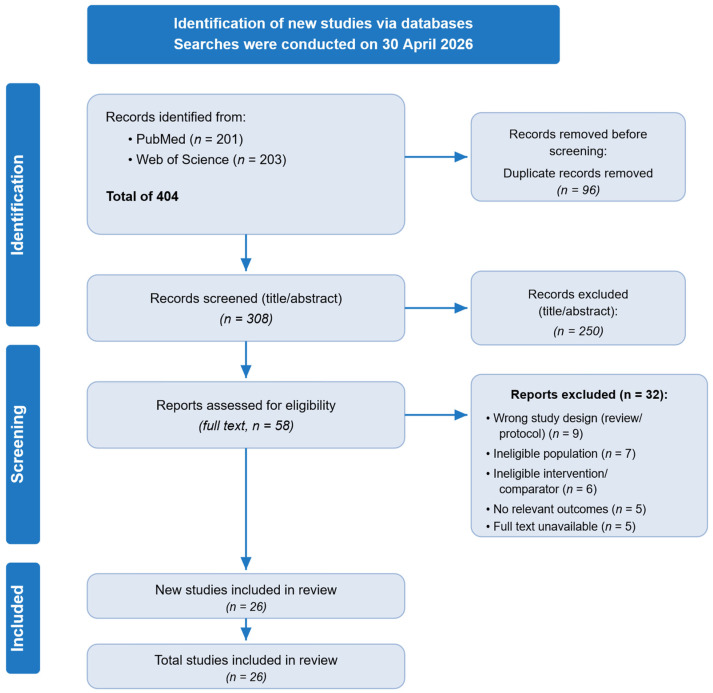
PRISMA 2020 flow diagram of the literature search and screening.

Screening proceeded in two stages: two reviewers first independently screened records by title and abstract, then retrieved the full texts of potentially eligible reports and independently re-screened them against the criteria in Table 1; disagreements at either stage were resolved by discussion and, where necessary, by adjudication from a third senior reviewer. Multiple reports of the same study (for example, a conference abstract and the full paper) were linked and counted as a single study to avoid double-counting. The number of records, the number of exclusions, and the specific full-text exclusion reasons at each screening stage are shown in the PRISMA flow diagram (Figure 1).

### 2.5. Risk of Bias and Methodological Quality Assessment

Two reviewers independently assessed the risk of bias of the included studies, resolving disagreements by discussion or third-party adjudication. Because all included studies were randomized (double- or single-blind) placebo-controlled crossover trials, the crossover version of the Cochrane Risk of Bias 2 (RoB 2) tool was used throughout [20], focusing on the randomization process, the period and carryover effects specific to crossover designs (and whether the washout period was adequate), deviations from the intended intervention and blinding integrity, missing outcome data, measurement of the outcome, and selection of the reported result. Domain-level ratings were aggregated according to the established algorithm, and an overall judgment (low risk/some concerns/high risk) was assigned by outcome category or key outcome. In addition, methodological quality was assessed with the PEDro scale (11 items, of which item 1 concerns eligibility criteria and is not counted toward the total, giving a maximum of 10 scored points) [21], scored independently by two reviewers with disagreements resolved by discussion. Study-level RoB 2 overall ratings and designs, together with outcome-level judgments, are provided in Supplementary Tables S3 and S4.

### 2.6. Data Extraction and Management

Data were extracted using a standardized Microsoft Excel form that was developed and piloted before formal extraction. Two reviewers extracted the data independently and cross-checked all entries. Disagreements were resolved by returning to the original article and, when necessary, discussion between reviewers. The extracted information included bibliographic details, participant characteristics, sport type, competitive level, sample size, habitual caffeine intake, intervention characteristics, caffeine formulation or source, absolute and relative dose, timing of intake before competition or testing, blinding, study design, outcome measures, and the condition-specific sample size, mean, and standard deviation for caffeine and placebo. Female-specific variables were extracted where available, including age, menstrual-cycle phase, method of menstrual-cycle verification, hormonal-contraceptive use, and any reported control of cycle-related symptoms or testing phase. Habitual caffeine intake was extracted as reported in the original studies. For synthesis and moderator coding, habitual intake was classified at the review level as low, moderate, high, or unknown based on the quantitative intake values or descriptive categories provided in each primary study. When the original report did not provide sufficient information to support classification, habitual intake was coded as unknown. These classifications were used only for descriptive purposes and exploratory moderator analyses. Information on blinding checks, participants’ ability to identify the caffeine or placebo condition, and adverse symptoms or side effects was also extracted where reported. When adverse symptoms were not assessed or not reported in the original study, this was not interpreted as evidence that no symptoms occurred. Data standardization and missing data management followed prespecified rules. When standard errors were reported, standard deviations were derived using the reported sample size. When confidence intervals were reported, standard deviations were derived from the available interval information when this was compatible with the study design and reporting format. For data presented only in figures, two reviewers independently digitized the values using WebPlotDigitizer 4, and their readings were averaged to reduce extraction error.

For outcomes assessed repeatedly at multiple time points, each eligible post-supplementation time point was extracted as a dependent effect size. Change scores were not modeled separately. Dependence among repeated effects from the same study was handled during the meta-analysis. For studies with multiple caffeine dose arms, each dose arm was extracted separately and matched to the corresponding placebo condition. For the studies by Karayigit and colleagues, Bougrine and colleagues, and Quan and colleagues, the relative dose corresponding to each effect size was reconstructed from the original report. For the energy drink study by Arazi and colleagues, the relative caffeine dose was estimated from the reported drink volume and caffeine concentration. Multiple caffeine arms that shared the same placebo condition were identified at the effect size level and accounted for in the dependence structure during analysis. When required data were incomplete, corresponding authors were contacted by email to request missing information.

Each extracted effect size was linked to the corresponding study, caffeine dose arm, placebo condition, outcome domain, outcome submetric, and post-supplementation time point where applicable. We also recorded whether an effect size came from multiple outcomes within the same study, repeated post-supplementation assessments, multiple caffeine dose arms, or comparisons sharing the same placebo condition. These features were used to define the study-level clustering and working within-study dependence structure in the meta-analysis. No included comparison involved caffeine combined with an independent co-intervention; caffeine-containing products such as coffee, energy drinks, chewing gum, or capsules were treated as different caffeine sources or formulations.

Outcomes were classified a priori as physical performance, sport-specific performance, physiological responses, perceptual responses, or cognitive performance, and their direction was coded during extraction: higher values indicated more favorable performance for measures such as strength, power, jump height, running speed or distance, ball velocity, accuracy, and advantageous perceptual ratings, whereas lower values indicated more favorable performance for time-based tests, fatigue indices, ratings of perceived exertion, perceived pain or fatigue, and reaction time; these were reversed in sign before synthesis. Physiological outcomes (e.g., heart rate, blood lactate, blood glucose) were also direction-aligned, using for each indicator the direction conventionally regarded as favourable in exercise-physiology practice (lower heart rate, lower blood lactate, lower blood glucose). A positive effect size therefore denotes a shift in the conventionally favourable direction and a negative effect size a shift in the opposite direction. We emphasise that this is an analytic convention only: these indicators differ in clinical desirability and are interpreted as physiological response patterns rather than as unambiguous benefit or harm. For transparency, the sign applied to every physiological effect size, together with its unsigned value, is provided in the effect-size coding file deposited on OSF.

The complete effect-size-level extraction and coding datasets is available on the Open Science Framework on 21 May 2026 (OSF; https://osf.io/6hqfp, 10.17605/OSF.IO/QZYWT), allowing each modelled effect size to be traced to its source study, comparison structure, intervention characteristics, outcome classification, time point, direction coding, and condition-level data used for effect-size calculation.

### 2.7. Statistical Analysis

For each outcome, the effect size was expressed as the standardized mean difference between the caffeine and placebo conditions and reported as Hedges g. Two sources of dependence were distinguished. The first was the within-participant pairing inherent to crossover trials, in which the same athletes completed caffeine and placebo conditions. Because paired standard deviations, change-score standard deviations, or within-participant caffeine–placebo correlations were rarely reported, this source of dependence could not be fully incorporated into the primary effect-size variance. Therefore, the primary analysis estimated Hedges’ g from condition-level means and standard deviations, and the precision of individual study-level standard errors and confidence intervals should be interpreted as approximate.

The second source was the dependence among multiple effect sizes contributed by the same study, arising from multiple outcomes, repeated post-supplementation time points, multiple caffeine dose arms, or shared placebo comparisons. This source of dependence was addressed using a correlated and hierarchical effects three-level model with study-level clustering and CR2 robust variance estimation. Effect sizes were nested within studies, and a working sampling-error correlation of ρ = 0.6 was specified for effect sizes from the same study, including comparisons sharing the same placebo condition. Sensitivity analyses using alternative working within-study correlations (ρ = 0.0–0.9; reported at ρ = 0.5, 0.7, and 0.9 in Supplementary Table S6) were used to examine whether the pooled conclusions were robust to this assumption. Effect sizes and sampling variances were computed in R version 4.5.3 using a reproducible script consistent with the standardized mean difference option in the metafor package version 5.0-1.

Outcomes for physical performance, sport-specific performance, and perceptual responses were directionally aligned so that positive values indicated a favorable effect of caffeine. Measures for which lower values indicate better performance, such as sprint time or perceived exertion, were reversed before synthesis. Physiological outcomes, including heart rate, blood lactate, and blood glucose, were direction-aligned on the same convention (lower = favourable) and interpreted as physiological response patterns rather than as uniformly beneficial or harmful effects. Effect size magnitudes were interpreted using Cohen’s descriptive benchmarks as trivial, small, moderate, or large. These thresholds were used only to aid interpretation and were not treated as fixed biological cut points. Each effect size was also assigned to one of the prespecified outcome domains and labeled with its specific subindicator. Because most studies reported multiple dependent effect sizes, pooled analyses used a correlated and hierarchical effects three-level working model. Effect sizes were nested within studies, allowing the model to distinguish variation among studies from variation among effects within the same study. The primary analysis assumed a within-study working correlation of ρ = 0.6 among the sampling errors of effect sizes contributed by the same study, including comparisons that shared a common placebo condition. Because paired standard deviations, change-score standard deviations, and within-participant caffeine–placebo correlations were rarely reported, the sampling variance of each caffeine–placebo contrast was computed with the standard independent-groups formula for Hedges’ g, v = 1/n_C + 1/n_P + g^2^/[2(n_C + n_P)], with the small-sample correction factor J applied; this is a conservative approximation that does not exploit the paired structure of the crossover design. Within-study dependence was then handled through the block-diagonal working covariance matrix implied by ρ, so that inference does not rely on the working correlation being correctly specified. Sensitivity analyses repeated all models across ρ = 0.0–0.9. Variance components were estimated using restricted maximum likelihood. Robust variance estimation was applied with study as the clustering unit to reduce sensitivity to misspecification of the working dependence structure. Bias reduced linearization and Satterthwaite-approximated degrees of freedom were used for statistical inference. Pooled estimates, meta-regression coefficients, between-group contrasts, and confidence intervals were calculated using this robust framework. When the number of study clusters or the available degrees of freedom was small, the corresponding inferences were interpreted cautiously.

Residual heterogeneity was assessed using a model-based heterogeneity test, with a probability value below 0.10 considered statistical evidence of remaining heterogeneity. Multilevel heterogeneity estimates were reported, including their decomposition into within-study and between-study components. Prediction intervals were calculated to describe the range in which the true effect of a future comparable study might plausibly fall. Three-level and conventional two-level random-effects models were compared using information criteria and, when appropriate, likelihood ratio tests. When a variance component was estimated at the boundary, model comparison results were treated as diagnostic rather than definitive, and the implications for heterogeneity interpretation were reported transparently.

Exploratory subgroup analyses were conducted by outcome domain, dose category, formulation or source, sport, competitive level, habitual caffeine intake, and blinding. Dose categories were defined as low, moderate, and high according to relative caffeine intake per kg body mass. Each subgroup analysis refitted the same correlated and hierarchical effects model with robust variance estimation on the relevant subset. Between-group moderation was assessed using dummy-coded meta-regression and robust Wald tests. Continuous moderators, including relative dose, timing of intake, sample size, publication year, and ordinal habitual caffeine intake, were examined using meta-regression after mean centering. Both linear and quadratic dose models were fitted to explore possible nonmonotonic dose–response patterns.

A subgroup level or moderator was formally analyzed only when it included at least three studies. Otherwise, the result was presented descriptively. All subgroup and meta-regression analyses were considered exploratory because they were based on study-level characteristics, uneven numbers of studies across categories, relatively small numbers of study clusters for several moderator levels, and multiple comparisons; accordingly, individual nominally significant subgroup or sub-metric results may reflect chance, and were not treated as confirmatory. Subgroup-specific pooled estimates were reported to describe the distribution of available evidence, but they were not interpreted as evidence of moderation unless supported by a formal between-subgroup moderator test or robust meta-regression coefficient. Therefore, a statistically detectable effect within one subgroup, or a numerically larger point estimate in one subgroup than another, was not interpreted as evidence that caffeine effects differed by dose, timing of intake, formulation, habitual caffeine intake, sport, competitive level, blinding status, or population characteristics. Non-significant moderator tests were also not interpreted as evidence that these variables have no biological or practical relevance; rather, they were interpreted as indicating that the current evidence was insufficient to establish reliable moderation. These analyses were therefore treated as hypothesis-generating rather than confirmatory.

When at least ten studies were available for an outcome domain, funnel plot asymmetry was examined using an Egger-type regression that incorporated within-study dependence. A probability value above 0.05 was interpreted as showing no statistical evidence of small-study effects. No formal asymmetry test was conducted when fewer than ten studies were available. When asymmetry was detected, PET PEESE was applied as an exploratory bias correction.

The robustness of the main conclusions was examined using four sensitivity approaches. First, the assumed within-study working correlation was varied across a plausible range (ρ = 0.0–0.9) around the primary value of ρ = 0.6. Fourth, a conservative reduction analysis was performed in which each study contributed exactly one effect size per outcome domain: dependent effect sizes within a study and domain were combined by variance-weighted aggregation of correlated estimates (compound-symmetric structure, ρ = 0.6) and the aggregated data were then synthesized with a conventional two-level random-effects model, so that studies contributing many effect sizes could not exert disproportionate influence. Second, leave-one-study-out analyses were conducted using study as the removal unit. Third, outlier and influence diagnostics were examined using studentized residuals, leverage values, and Cook’s distance, followed by model refitting after removal of flagged observations. To describe the detection capacity of the included studies, statistical power was estimated for small and moderate effects and visualized by outcome domain using sunset funnel plots. This power analysis used a condition-level independent-groups approximation and was interpreted only as a conservative descriptive assessment. All analyses were performed in R, including the metafor package.

### 2.8. Certainty of Evidence (GRADE)

Two researchers independently assessed the certainty of evidence for each major outcome using the GRADE framework. The evidence was subsequently evaluated outcome by outcome and downgraded, where appropriate, for risk of bias, inconsistency, indirectness, imprecision, and publication bias. Downgrading decisions were based on an overall assessment of the evidence within each domain rather than on any single statistical threshold or individual study. Disagreements were resolved through discussion, with consultation of a third researcher when necessary [22].

## 3. Results

### 3.1. Literature Screening

After database searching, backward citation tracking, deduplication, title/abstract screening, and full-text assessment, 26 studies were included. The two electronic databases retrieved 404 records in total, including 201 from PubMed/MEDLINE and 203 from Web of Science. After removal of 96 duplicates, 308 records were screened by title and abstract, of which 250 were excluded. Fifty-eight reports were assessed in full text, and 32 were excluded for the following reasons: wrong study design, ineligible population, ineligible intervention or comparator, no relevant extractable outcomes, or unavailable full text. No report was excluded solely because of language after full-text assessment. The full selection process is shown in Figure 1.

### 3.2. Characteristics of Included Studies

**Figure 2:**
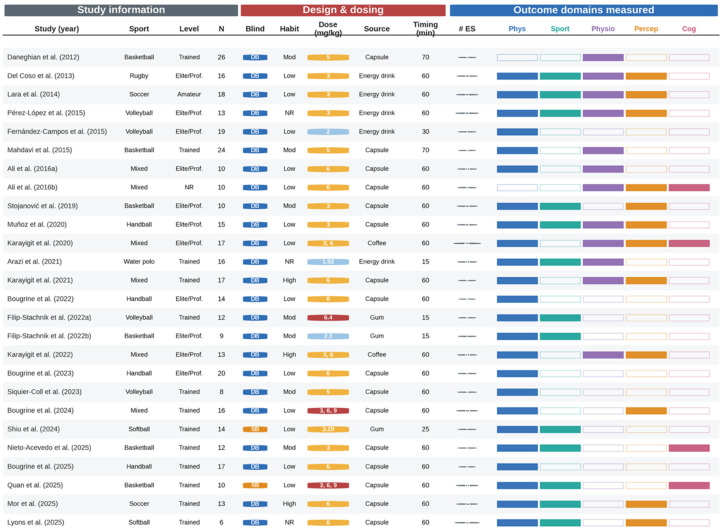
Overview of the characteristics of the included studies (design, participants, caffeine protocol, and outcome coverage). The figure summarizes the study design, sport, competitive level, sample size, caffeine source and dose, timing of pre-competition or pre-test intake, and the distribution of measured outcome domains across the 26 included studies. Note: N is the sample size reported by the study; for multi-dose-arm studies, the relative doses are listed separated by commas. All studies were placebo-controlled crossover designs [23,24,25,26,27,28,29,30,31,32,33,34,35,36,37,38,39,40,41,42,43,44,45,46,47,48].

The main characteristics of the included studies are summarized in Supplementary Table S2. Of the 26 studies, 24 used a randomized double-blind placebo-controlled crossover design and 2 used a single-blind design [23,24,25,26,27,28,29,30,31,32,33,34,35,36,37,38,39,40,41,42,43,44,45,46,47,48]. The sports covered basketball (6 studies), mixed team ball sports (6 studies), volleyball (4 studies), handball (4 studies), soccer (2 studies), softball (2 studies), and rugby and water polo (1 study each); competitive level included elite/professional (11 studies), well-trained/sub-elite (13 studies), and amateur/recreational (1 study), with 1 study not clearly reporting. Caffeine was delivered mainly as capsules or anhydrous caffeine (16 studies), with the remainder as energy drinks (5 studies), coffee (2 studies), and chewing gum (3 studies); doses ranged from 1.92 to 9 mg·kg^−1^, with 4 studies using multiple dose arms, and the median timing of pre-competition or pre-test intake was 60 min (range 15–70 min). In terms of outcome coverage, 24 studies reported physical performance, 13 reported sport-specific performance, 12 reported physiological responses, 12 reported perceptual responses, and 4 reported cognitive performance. Reporting of female-specific variables was limited and inconsistent across the included studies. Menstrual-cycle phase and hormonal-contraceptive use were not reported or controlled with sufficient consistency to support reliable subgroup or meta-regression analyses. Habitual caffeine intake was more commonly reported than menstrual-cycle phase or hormonal-contraceptive use, but the reporting format varied across studies; therefore, it was coded descriptively as low, moderate, high, or unknown based on the information available in each primary report. Reporting of blinding checks and adverse symptoms was also inconsistent. Therefore, lack of reported adverse symptoms was treated as unclear unless the primary study explicitly stated that adverse events or side effects were assessed and absent. Detailed characteristics of the included studies are shown in Supplementary Table S2 and Figure 2.

### 3.3. Risk of Bias and Methodological Quality

Using the crossover version of the Cochrane Risk of Bias 2 tool, none of the 26 included studies was judged to be at overall low risk of bias. Twenty-three studies were judged as raising some concerns, and three studies were judged to be at high risk of bias, namely Filip-Stachnik et al. 2022 [46], Bougrine et al. 2025 [38], and Quan et al. 2025 [39]. The most frequent concerns related to insufficient reporting of the randomization process, including sequence generation and allocation concealment, and to the selection of the reported result. For crossover trials, additional concerns related to period effects, carryover effects, washout adequacy, and blinding integrity were also considered. The high-risk judgments were mainly driven by the deviations from the intended intervention domain, where the perceptible effects of caffeine may have compromised blinding and influenced participant behavior or effort during testing. This concern was considered relevant not only for subjective perceptual outcomes but also for effort-dependent physical or sport-specific tests performed at maximal or near-maximal intensity. PEDro scores ranged from 5 to 9, with a median of 7 out of 10, suggesting generally adequate reported trial conduct. However, PEDro was treated as supplementary methodological quality information rather than as a substitute for the RoB 2 risk of bias assessment. Study-level RoB 2 overall judgments are presented in Supplementary Table S4. Full domain-level RoB 2 judgments and justifications are provided in Supplementary Table S4, and study-level PEDro item scores are provided in Supplementary Table S3.

### 3.4. Main Pooled Analysis

**Figure 3:**
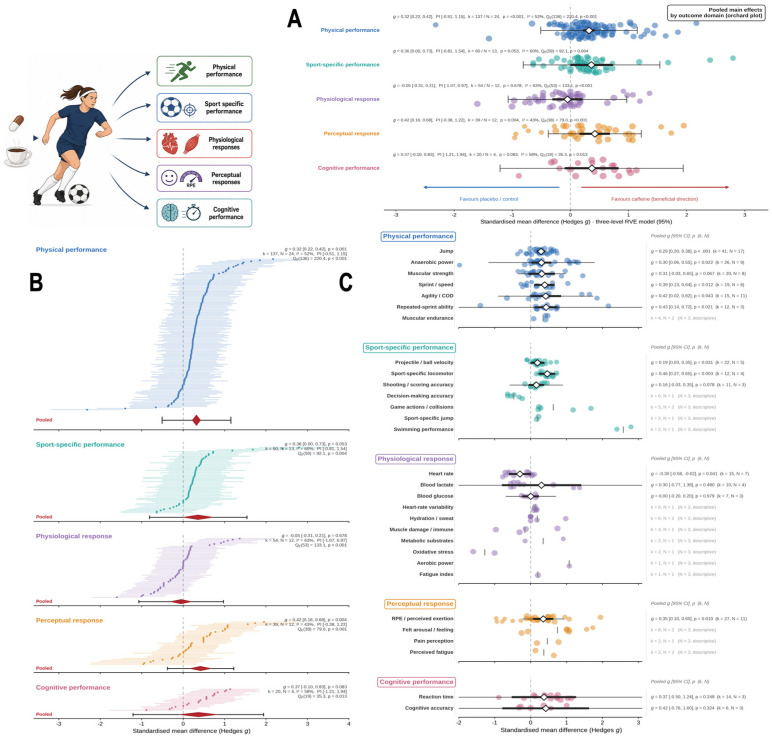
Orchard plot of the acute effect of caffeine on each outcome domain in female team-sport athletes. (**A**) Pooled main effects across the five outcome domains; (**B**) ordered individual effect-size estimates and pooled estimates for each domain; (**C**) pooled estimates for outcome submetrics within each domain. Effect sizes are standardized mean differences expressed as Hedges’ g (caffeine versus placebo), computed from condition-level means and within-condition standard deviations with the small-sample correction applied. White diamonds are the pooled estimates from a three-level correlated-and-hierarchical-effects (CHE) model, in which effect sizes are nested within studies and a within-study working correlation of ρ = 0.6 is assumed among effect sizes from the same study, including comparisons that share a placebo condition; inference uses CR2 cluster-robust variance estimation with study as the clustering unit and Satterthwaite degrees of freedom. Thick lines are 95% confidence intervals, thin whiskers are 95% prediction intervals, individual bubbles are the contributing effect sizes, and bubble area is proportional to precision (1/SE). The dashed line is the null line; positive values indicate a direction favorable to caffeine. Physiological responses are presented on the direction-aligned scale, using the direction conventionally regarded as favourable for each indicator (lower heart rate, lactate, and glucose); a negative value therefore denotes an increase in the indicator after caffeine (e.g., a higher heart rate). Because this domain aggregates indicators of differing clinical desirability, its pooled value is descriptive only and should not be read as uniform benefit or harm. The number of effect sizes (k) and of contributing studies (*N*) is given for each domain: physical performance k = 137, *N* = 24; sport-specific performance k = 60, *N* = 13; physiological responses k = 54, *N* = 12; perceptual responses k = 39, *N* = 12; cognitive performance k = 20, *N* = 4. Because the number of independent studies, rather than the number of effect sizes, determines the effective sample size under cluster-robust inference, domains supported by few studies, notably cognitive performance (*N* = 4, robust *df* ≈ 2.6)—carry wide intervals and should be regarded as preliminary. The cross-domain (omnibus) estimate is retained only as a descriptive summary, because the five domains do not represent a single underlying construct; the domain-specific estimates are the primary interpretable findings. Full model statistics for every estimate shown here are provided in Supplementary Table S5, and sub-metric estimates in Supplementary Table S7. CHE, correlated-and-hierarchical effects; CI, confidence interval; PI, prediction interval; SE, standard error; ρ, within-study working correlation.

Across all available domains, including direction-aligned physiological responses, the pooled omnibus effect of acute caffeine was small: g = 0.24, 95% CI [0.13, 0.35], *p* < 0.001; Q_E [*df* = 309] = 631.1, *p* < 0.001; *I*^2^ = 59.0%; 95% PI [−0.70, 1.18]; Supplementary Table S5. This omnibus estimate was retained only as a descriptive cross-domain summary because the included domains do not represent a single underlying biological or performance construct. It also combines heterogeneous outcome types and is weighted toward the more numerous physical-performance effects. Therefore, the domain-specific estimates below were treated as the primary interpretable findings. Domain-specific pooled results are shown in Figure 3, and full main-effect model statistics are provided in Supplementary Table S5. In Figure 3, physiological responses are plotted on the direction-aligned scale (positive = conventionally favourable), but because the domain aggregates indicators of differing clinical desirability, its pooled value should be read as descriptive only.

#### 3.4.1. Physical Performance

The pooled effect for physical performance was small and statistically significant (g = 0.32, 95% CI [0.22, 0.42], *p* < 0.001; Q_E [*df* = 136] = 220.4, *p* < 0.001; *I*^2^ = 52.2%, level-3 variance estimated at 0; 95% PI [−0.51, 1.15]; Supplementary Table S5). For the specific indicators within this domain, caffeine showed a small favorable direction with 95% confidence intervals that did not cross the null for repeated-sprint ability (g = 0.43, 95% CI [0.14, 0.72], *p* = 0.021; k = 12, studies = 3), agility/change in direction (g = 0.42 [0.02, 0.82], *p* = 0.043; k = 15, studies = 11), sprint/speed (g = 0.39 [0.13, 0.64], *p* = 0.012; k = 19, studies = 6), anaerobic power (g = 0.30 [0.06, 0.55], *p* = 0.022; k = 26, studies = 9), and jumping (g = 0.29 [0.20, 0.38], *p* < 0.001; k = 41, studies = 17); the point estimate for muscular strength was in the small-effect direction but its confidence interval crossed the null (g = 0.31 [−0.03, 0.65], *p* = 0.067; k = 20, studies = 8), so the statistical evidence was insufficient; muscular endurance was not pooled because fewer than three studies were available (k = 4, studies = 2). The pooled effects for each sub-indicator are shown in Figure 3, and the full sub-metric results are provided in Supplementary Table S7.

#### 3.4.2. Sport-Specific Performance

Within this domain, outcomes were grouped into match-related external load (sport-specific locomotion/running, e.g., high-intensity running distance and pace), ball-speed outcomes, and skill-accuracy outcomes, because high-intensity running and pace reflect external match load rather than technical skill. The pooled point estimate for sport-specific performance suggested a small-effect direction, but the 95% confidence interval crossed the null and the statistical evidence was insufficient (g = 0.36, 95% CI [−0.01, 0.73], *p* = 0.053; Q_E [*df* = 59] = 92.1, *p* = 0.004; *I*^2^ = 60.2%, of which 58.2% was at the between-study level and only 2.1% within studies; Supplementary Table S5), with heterogeneity arising mainly from between-study differences. For the specific indicators, sport-specific locomotion/running performance (such as high-intensity running distance and pace; g = 0.46 [0.27, 0.65], *p* = 0.003; k = 12, studies = 4) and throwing/ball-speed indicators (pitch speed, batted-ball exit velocity, spike speed, etc.; g = 0.19 [0.03, 0.35], *p* = 0.031; k = 22, studies = 5) showed a favorable direction, although each of these sub-metrics was based on only a few studies; the confidence interval for shooting/scoring accuracy crossed the null (g = 0.16 [−0.03, 0.35], *p* = 0.078; k = 11, studies = 3); decision-making accuracy, sport-specific jumping, contact actions, and swimming were not pooled because too few studies were available. These sub-metric results are shown in Figure 3 and Supplementary Table S7.

Because the domain-level estimate was borderline and its confidence interval crossed the null, whereas the favorable locomotion/running and ball-speed sub-metrics were each based on only four and five studies, respectively, these sub-metric findings should be regarded as exploratory and limited by the small number of contributing studies, and should not be interpreted as evidence that caffeine improves sport-specific performance overall.

#### 3.4.3. Physiological Responses

Physiological responses were direction-aligned (lower heart rate, lactate, and glucose coded as favourable) and interpreted as physiological response patterns rather than as beneficial or harmful performance effects. The pooled estimate across physiological variables did not show a clear domain-level direction (g = −0.05, 95% CI [−0.31, 0.21], *p* = 0.678; Q_E [*df* = 53] = 133.1, *p* < 0.001; *I*^2^ = 62.8%; 95% PI [−1.07, 0.97]; Supplementary Table S5); because this domain comprises distinct physiological indicators such as heart rate, blood lactate, and blood glucose, this pooled value serves only as a descriptive summary and should not be interpreted as the increase or decrease in a single physiological construct. For the specific indicators, the heart rate submetric showed a statistically detectable difference (g = −0.30 [−0.58, −0.02], *p* = 0.041; k = 15, studies = 7); the pooled effects for blood lactate (g = 0.30 [−0.77, 1.38], *p* = 0.480; k = 10, studies = 4) and blood glucose (g = 0.00 [−0.20, 0.20], *p* = 0.979; k = 7, studies = 3) were not statistically significant; heart rate variability, hydration/sweating, muscle damage/immune markers, oxidative stress, metabolic substrates, and aerobic power were not pooled because too few studies were available. These physiological sub-metric results are shown in Figure 3 and Supplementary Table S7.

#### 3.4.4. Perceptual Responses

The pooled effect for perceptual responses was small and statistically significant (g = 0.42, 95% CI [0.16, 0.68], *p* = 0.004; Q_E [*df* = 38] = 79.0, *p* < 0.001; *I*^2^ = 43.1%; 95% PI [−0.38, 1.22]; Supplementary Table S5). This effect was driven mainly by the rating of perceived exertion (RPE): caffeine showed statistical evidence of reducing perceived exertion during exercise tasks (g = 0.35 [0.10, 0.60], *p* = 0.010, with positive values indicating a reduction in RPE; k = 27, studies = 11); felt arousal/pleasure, perceived pain, and perceived fatigue were not pooled because too few studies were available. These perceptual sub-metric results are shown in Figure 3 and Supplementary Table S7.

#### 3.4.5. Cognitive Performance

The pooled point estimate for cognitive performance suggested a small-effect direction but did not reach statistical significance (g = 0.37, 95% CI [−0.10, 0.83], *p* = 0.083; Q_E [*df* = 19] = 35.3, *p* = 0.013; *I*^2^ = 57.7%; 95% PI [−1.21, 1.94]; Supplementary Table S5); with robust-inference degrees of freedom of only 2.6 and a basis of just four studies, the stability of the *p* value and the estimate is very limited. For the specific indicators, neither reaction time (g = 0.37 [−0.50, 1.24], *p* = 0.248) nor cognitive accuracy (g = 0.42 [−0.76, 1.60], *p* = 0.324) reached significance. These cognitive sub-metric results are shown in Figure 3 and Supplementary Table S7.

### 3.5. Moderator Analysis

Because several subgroups were informed by only a small number of studies, in some cases a single study or as few as three study clusters, all moderator and subgroup results should be interpreted with particular caution, as estimates based on so few studies are statistically unstable and readily influenced by individual trials.

**Figure 4:**
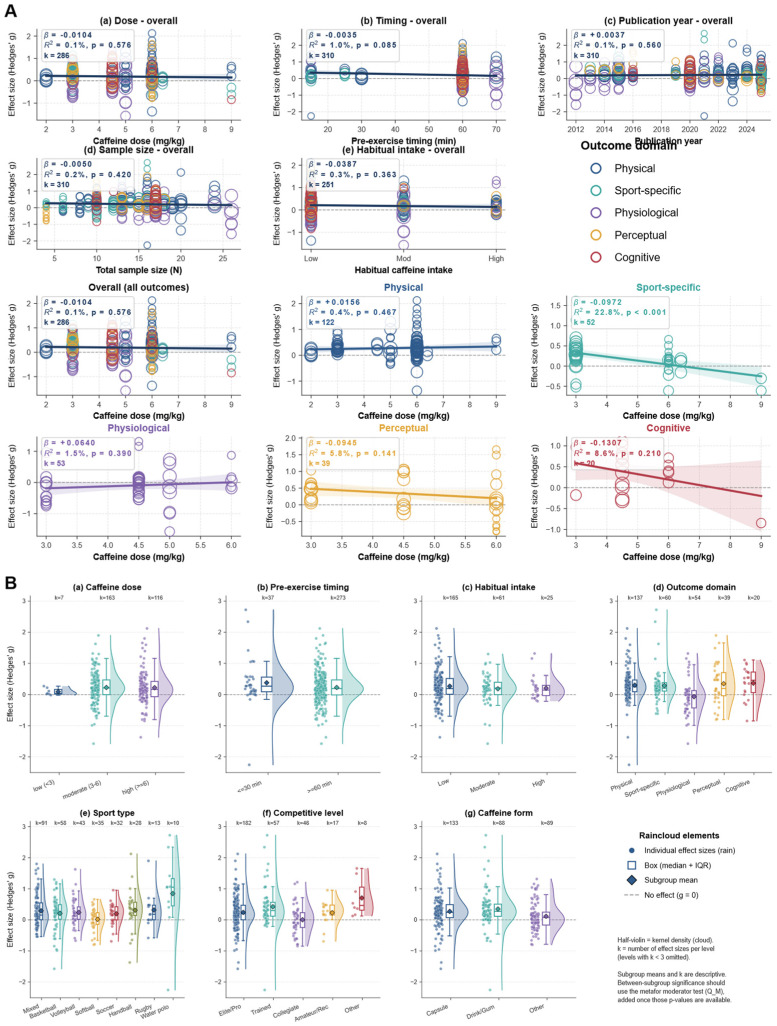
Exploratory moderator analysis of the caffeine effect on performance and physiological outcomes in female team-sport athletes. Effect sizes are Hedges’ g; positive values favor caffeine over placebo. Upper composite: continuous-moderator meta-regressions. (**A**) Overall weighted meta-regressions of g on caffeine dose (**a**), pre-exercise timing (**b**), publication year (**c**), total sample size (**d**), and the ordinal measure of habitual intake (**e**). (**B**) Dose–response meta-regressions fitted separately within each outcome domain. In every panel, each bubble is one effect size, sized by its inverse-variance weight (1/SE^2^) and colored by outcome domain; the solid line is the linear meta-regression fit with its 95% confidence band, and the dashed line marks the null (g = 0). Slope (β), R^2^, and *p* values are annotated per panel. Lower composite: subgroup rainclouds. Panels (**a**–**g**) show the distribution of g across levels of each categorical moderator: caffeine dose category (**a**), pre-exercise timing (**b**), habitual intake (**c**), outcome domain (**d**), sport type (**e**), competitive level (**f**), and caffeine source (**g**). For each level, individual effect sizes (jittered points, “rain”) are shown beside a box plot representing the median and interquartile range and a half-violin kernel density estimate (“cloud”), with the filled diamond denoting the subgroup mean; levels with fewer than three effect sizes are omitted. Subgroup means and k are descriptive; between-group differences were tested with robust Wald tests in the multilevel model and were non-significant for all moderators (*p* > 0.05). Therefore, these visual subgroup patterns should be interpreted as exploratory and hypothesis-generating only, and not as evidence of dose-, timing-, habitual-intake-, sport-, competitive-level-, or caffeine-source-specific effects. Crossover sampling variances were computed assuming a within-study working correlation of ρ = 0.6, the value used in the primary model.

Exploratory analyses were conducted for each continuous and categorical moderator. The meta-regression slopes for dose (overall β = +0.023, *p* = 0.362), timing of intake (β = −0.003, *p* = 0.446), sample size (β = −0.006, *p* = 0.619), publication year (β = +0.016, *p* = 0.383), and the ordinal measure of habitual intake (β = −0.075, *p* = 0.232) were all non-significant, and each remained so when fitted separately within every outcome domain (Figure 4; Supplementary Tables S9.1 and S9.2). The between-group robust Wald tests for the categorical moderators (dose category, caffeine source, sport, competitive level, and habitual intake) were likewise all non-significant (*p* > 0.05), both overall and within each domain; full statistics for the overall and domain-specific subgroup models are provided in Supplementary Tables S8.1–S8.6.

Because effect sizes were distributed unevenly across moderator levels, we additionally inspected the within-moderator subgroup estimates descriptively. For dose category, the commonly studied moderate-dose range showed favorable within-subgroup estimates overall (g = 0.22, *p* = 0.001) and for physical (g = 0.32), perceptual (g = 0.43), and cognitive (g = 0.45) outcomes. However, the low-dose category was based on a single study, and the high-dose category did not show a statistically detectable within-subgroup estimate after accounting for the multilevel structure. Because the formal between-dose moderation test was non-significant, these descriptive differences should not be interpreted as evidence that one dose category is superior to another.

Descriptively, subgroup estimates by habitual caffeine intake showed numerically larger favorable effects in studies classified as low habitual intake than in those classified as high habitual intake. In low-habitual-consumer studies, the pooled estimates were favorable for the overall analysis (g = 0.26), physical performance (g = 0.34), and perceptual responses (g = 0.53). For physical performance, the estimate was g = 0.34 (95% CI 0.19 to 0.50, *p* < 0.001, k = 67, studies = 12) in the low habitual-intake subgroup and g = 0.11 (95% CI −0.06 to 0.27, k = 18, studies = 3) in the high habitual-intake subgroup. However, the formal between-subgroup moderation test was not statistically significant (*p* = 0.096), and the high-intake subgroup included only three study clusters. Therefore, these subgroup findings should not be interpreted as evidence that habitual caffeine intake or caffeine dose modifies the ergogenic effect of caffeine. They are reported only as exploratory, hypothesis-generating observations for future adequately powered trials with prespecified moderator analyses.

### 3.6. Publication Bias and Sensitivity Analysis

**Figure 5:**
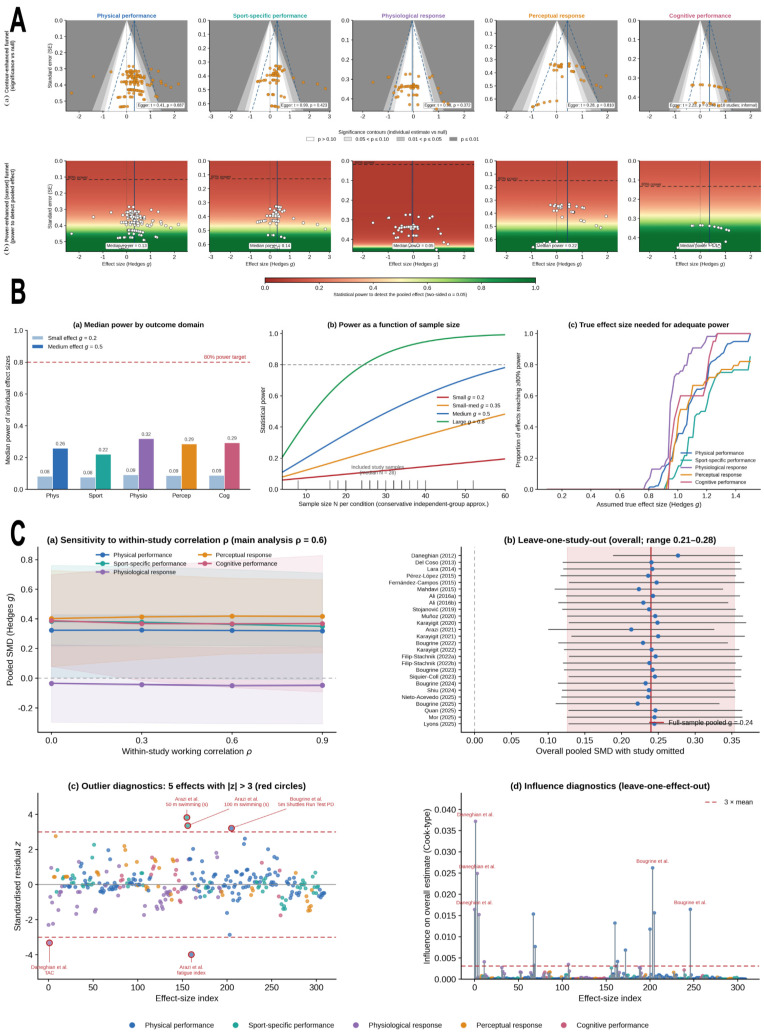
Publication bias, statistical power, and sensitivity analyses across the five outcome domains. Note: (**A**) Contour-enhanced funnel plots (**a**) and power-enhanced “sunset” funnel plots (**b**). In the contour plots, each point is an effect size (Hedges’ g) against its standard error; shaded bands mark significance contours (*p* < 0.10, <0.05, <0.01) and the dashed line the pooled estimate. Egger-type regression did not detect statistically significant asymmetry in any domain (overall intercept = 4.13, *p* = 0.106; Supplementary Table S10.1). Because the evidence base comprised few, small, and partly dependent studies, this finding indicates that publication bias was undetected but not excluded; it does not demonstrate the absence of publication bias. Sunset plots color-code each study’s power (green = adequate, red = underpowered), highlighting the predominance of small, low-power studies. (**B**) Power diagnostics. (**a**) Median power per domain to detect a small effect (g = 0.2) at α = 0.05 (~8–9%). (**b**) Power versus per-group sample size for assumed true effects of g = 0.2–1.0; dashed line = 80%. (**c**) True effect size required for 80% power, by domain. Power to detect a moderate effect (g = 0.5) reached only ~22–32%, and no effect size attained 80%. (**C**) Sensitivity and influence analyses of the pooled estimate. (**a**) Sensitivity to the within-study correlation ρ (0.0–0.9; main analysis ρ = 0.6); estimates were essentially invariant to ρ (all-domain g = 0.23–0.24) [23,24,25,26,27,28,29,30,31,32,33,34,35,36,37,38,39,40,41,42,43,44,45,46,47,48]. (**b**) Leave-one-study-out (all-domain model): pooled SMD ranged 0.21–0.28. (**c**) Outlier diagnostics: five effect sizes exceeded |z| > 3 (red circles); their removal left the estimate unchanged at g = 0.24 [0.13, 0.34]. (**d**) Influence diagnostics (leave-one-effect-out, Cook’s distance); dashed line = 3 × mean. SMD, standardized mean difference; ρ, within-study working correlation. Effect sizes are Hedges’ g; colors denote the five outcome domains throughout. PET-PEESE was not applied given the few studies, dependence among effect sizes, and low power of the asymmetry test.

With respect to publication bias, the Egger-type regression for each outcome domain found no statistical evidence supporting funnel-plot asymmetry (overall: intercept = 4.13, *p* = 0.106; physical performance: intercept = 0.90, *p* = 0.687; sport-specific performance: intercept = 4.69, *p* = 0.423; physiological responses: intercept = 2.99, *p* = 0.372; perceptual responses: intercept = 1.60, *p* = 0.810; the full intercepts, SEs, *df*, *p* values, and cautionary note for cognitive performance are provided in Supplementary Table S10.1). However, given the limited number of studies per outcome, the dependence among effect sizes, and the predominance of small studies, these tests had limited power. Accordingly, a non-significant Egger-type test was interpreted as publication bias being “undetected but not excluded”, rather than as evidence of no publication bias; PET-PEESE correction was therefore not performed (Figure 5).

Sensitivity analyses showed that the main-effect estimates were robust. Relative to the primary model (ρ = 0.6), the all-domain pooled g was 0.244, 0.236, and 0.228 at within-study correlations of ρ = 0.5, 0.7, and 0.9, respectively, and the physical-performance estimate remained similarly stable (g = 0.323, 0.321, and 0.319 across the same ρ values; Supplementary Table S6). Leave-one-study-out analyses also suggested stability: the all-domain estimate ranged from 0.21 to 0.28, and the physical-performance estimate ranged from 0.30 to 0.34 (Supplementary Table S10.2). Outlier and influence diagnostics identified five effect sizes with |z| > 3, after whose removal the all-domain estimate remained essentially unchanged at g = 0.24 [0.13, 0.34] (Supplementary Table S10.3). Finally, a conservative reduction analysis in which each study contributed exactly one effect size per outcome domain (variance-weighted aggregation of correlated estimates at ρ = 0.6, followed by a two-level random-effects model) reproduced the domain-level findings: physical performance g = 0.29 (95% CI 0.16 to 0.42), sport-specific performance g = 0.37 (0.03 to 0.70), physiological responses g = 0.00 (−0.24 to 0.24), perceptual responses g = 0.38 (0.18 to 0.57), and cognitive performance g = 0.34 (−0.01 to 0.69) (Supplementary Table S11). The pooled estimates were therefore not driven by studies contributing many effect sizes. The only change in inferential status was for sport-specific performance, which was borderline in the primary model (*p* = 0.053) and reached nominal significance in this reduced analysis (*p* = 0.034); because the reduced model discards the within-study structure that the primary model is designed to accommodate, we retain the more cautious primary interpretation for this domain.

The statistical power analysis showed that the median power of an individual effect size to detect a small effect (g = 0.2) at α = 0.05 was only about 8–9%, and only about 22–32% to detect a moderate effect (g = 0.5), with no effect size reaching 80% power, indicating that individual primary studies have very limited capacity to detect small-to-moderate effects (Figure 5).

### 3.7. Certainty of Evidence (GRADE)

Using the GRADE approach, each outcome domain was assessed across risk of bias, inconsistency, indirectness, imprecision, and publication bias (Table 2). For physical performance, certainty started at high and was downgraded one level for risk of bias because no included trial reached overall low risk of bias, although the predominant judgment was “some concerns” rather than “high risk.” We did not apply an additional downgrade for inconsistency: although between-study heterogeneity was moderate and the prediction interval was wide, the pooled point estimate was direction-consistent and stable across leave-one-out and influence analyses. Indirectness and imprecision did not warrant further downgrading because the directness of the population, intervention, and outcomes was appropriate and the confidence interval excluded the null. Publication bias was judged undetected but not excluded, without an additional downgrade. The final certainty rating was therefore moderate.

For sport-specific performance, certainty was downgraded one level for risk of bias, because no included trial reached overall low risk of bias, one level for inconsistency because of high between-study heterogeneity, and one level for imprecision because the confidence interval crossed the null, resulting in very low certainty. Indirectness was not judged serious, and publication bias was considered undetected but not excluded.

For physiological responses, certainty was downgraded one level for risk of bias, one level for inconsistency because the domain combined indicators with diverse biological meaning and showed high heterogeneity, one level for indirectness because this aggregate describes physiological response patterns rather than a single efficacy outcome, and one level for imprecision because the confidence interval crossed the null. The final rating was very low certainty. Publication bias was undetected but not excluded.

For perceptual responses, certainty was downgraded one level under the risk-of-bias domain. Caffeine can produce perceptible effects, including alertness, diuresis, tremor, and palpitations; blinding integrity may therefore have been compromised, which can bias subjective ratings such as perceived exertion in the expected direction. The prediction interval crossed the null, but this did not warrant an additional downgrade. The final rating was moderate certainty, and the downgrade is explicitly attributed to compromised blinding rather than to the subjective nature of the outcome itself.

For cognitive performance, certainty was downgraded one level for risk of bias, one level for inconsistency because of high heterogeneity, and two levels for imprecision because only four studies contributed data, robust degrees of freedom were approximately 2.6, and the confidence and prediction intervals were very wide. The final rating was very low certainty; these estimates should be interpreted as preliminary. Across all domains, a non-significant asymmetry test was not equated with the absence of publication bias, and the publication-bias judgment was consistently recorded as undetected but not excluded.

## 4. Discussion

This systematic review and meta-analysis synthesized 26 randomized, placebo-controlled crossover trials to estimate the acute effects of caffeine ingestion in female team-sport athletes across five outcome domains. In summary, caffeine produced a small but consistent benefit for physical performance and a reduction in perceived exertion, whereas the effects on sport-specific and cognitive outcomes remained uncertain, being borderline or non-significant and resting on few studies per outcome. Physiological responses showed no clear overall direction at the domain level, reflecting the aggregation of indicators with differing clinical desirability. Across all exploratory moderator analyses, no factor, including dose, timing of intake, formulation or source, sport type, competitive level, habitual caffeine intake, or blinding status, provided reliable evidence of moderation. Thus, the central message is one of a modest, domain-dependent ergogenic effect that is most defensible for physical performance and perceived exertion and should not be generalized to sport-specific, cognitive, or moderator-specific claims.

### 4.1. Physical Performance

Physical performance was the most densely studied domain and yielded the most stable conclusion: a small, significant, direction-consistent benefit (g = 0.32). Favorable effects with confidence intervals excluding the null were observed for repeated-sprint ability (g = 0.43), agility and change in direction (g = 0.42), sprint and speed (g = 0.39), anaerobic power (g = 0.30), and jumping (g = 0.29), the last resting on the largest evidence base (k = 41, 17 studies) and therefore the single most credible physical finding. This pattern is biologically plausible: as a non-selective adenosine-receptor antagonist, caffeine relieves central inhibition, increases motor-cortex excitability and motor-unit recruitment, and may improve excitation–contraction coupling and calcium handling, thereby enhancing maximal and repeated maximal neuromuscular output [49,50]. The direction is consistent with general-population meta-analyses reporting benefits for muscular strength and power [51,52], Wingate anaerobic performance [52], and repeated-sprint ability [53]. Repeated-sprint ability may be an especially suitable target, because it combines high neuromuscular output with fatigue tolerance across efforts and so benefits from a reduced perception of effort as fatigue accumulates [50,53]. The estimates should nonetheless be read through their sub-outcomes rather than as one homogeneous construct [10,11].

For jumping, the favorable direction is clearest for tasks relying on rapid force production and stretch–shortening-cycle function such as countermovement and vertical jumps, although the evidence is not uniform, since Gomez-Bruton et al. reported benefits for countermovement but not squat jump and Zhang et al. found no significant pooled jump effect in female basketball players despite a moderate point estimate [15,16]. For sprinting the benefit is smaller and less stable, with significant effects in mixed team-sport athletes but null or borderline effects in female-only samples, plausibly reflecting sprint distance, acceleration-phase dominance, timing method, and the limited sensitivity of short sprint tests to small ergogenic effects [11,16,54]. For agility, closed change-of-direction tests are more directly driven by sprint speed, braking, and lower-limb power, whereas reactive tasks also demand perception and decision-making, which may explain why favorable agility effects appeared in some syntheses but not in female basketball players [12,16]. By contrast, the point estimate for muscular strength was favorable but its confidence interval crossed the null (g = 0.31, *p* = 0.067), in contrast to the more stable intermittent-output indicators; this differs from broader strength meta-analyses that drew mainly on male or mixed-sex samples and more diverse tests, and in female team-sport athletes strength outcomes remain too sparsely and heterogeneously measured for a firm conclusion [51,52,55]. The GRADE rating for this domain was moderate after one downgrade for risk of bias, reflecting that no included trial was at overall low risk of bias. Although heterogeneity was moderate and the prediction interval was wide, the direction-consistent estimate was stable across leave-one-out and influence analyses; inconsistency was therefore not judged to warrant an additional downgrade. The applied interpretation is that caffeine may provide a modest benefit for explosive and repeated high-intensity actions, whereas its effect on maximal strength remains unresolved.

### 4.2. Sport-Specific Performance

At the domain level, the pooled sport-specific estimate was borderline and its confidence interval crossed the null, so the evidence does not support a general sport-specific benefit. Interpreted by construct rather than as a single technical-skill domain, the more favorable signals for locomotor/running and ball-speed outcomes rested on only a few studies each and should be treated as exploratory, whereas evidence for shooting or scoring accuracy remained insufficient; this domain was rated very low certainty. Thus, caffeine may plausibly support selected high-intensity or power-related sport-specific outputs, but this hypothesis requires confirmation in adequately powered trials and should not be read as improving technical accuracy or tactical skill execution.

Although the pooled effects were statistically small (g ≈ 0.3), effect-size magnitude should not be equated with practical irrelevance in competitive sport. Elite competition is frequently decided by margins far smaller than the average between-athlete variation captured by a standardized mean difference: sprints, jumps, and repeated high-intensity efforts are often separated by fractions of a second or a few centimetres, and a match can turn on a single decisive action. A group-level effect of g ≈ 0.3 therefore does not imply a trivial benefit for the individual athlete, but may correspond to a meaningful competitive advantage when applied to the specific, high-stakes actions that discriminate success in team-sport. This interpretation is reinforced by the accompanying reduction in perceived exertion, which may help athletes sustain these outputs for longer as fatigue accumulates. Accordingly, small ergogenic effects in this context are best judged against the smallest worthwhile change relevant to each competitive setting rather than against conventional statistical thresholds for a “small” effect.

### 4.3. Perceptual and Physiological Responses

Physiological outcomes were direction-aligned using the value conventionally regarded as favourable for each indicator, and interpreted as physiological response patterns rather than as benefit or harm. The pooled domain-level estimate did not show a clear direction (g = −0.05), reflecting that the domain aggregates indicators of differing clinical desirability. Heart rate was the only physiological sub-metric whose confidence interval excluded the null (g = −0.30 [−0.58, −0.02]; k = 15, 7 studies). On the direction-aligned scale, in which a lower heart rate is coded as favourable, this negative value indicates that caffeine increased heart rate. This is the expected pharmacological response to a non-selective adenosine-receptor antagonist and is consistent with caffeine’s known sympathoexcitatory and chronotropic effects; it should be read as a marker of pharmacological engagement rather than as an adverse event, since a modest rise in heart rate during exercise carries no established performance or safety penalty in healthy athletes. Notably, this cardiovascular response occurred alongside a reduction in perceived exertion, so athletes rated the same workload as easier despite a slightly higher heart rate, a dissociation consistent with a central rather than peripheral mechanism of action. Given the small number of contributing studies and a confidence interval lying close to the null, this estimate should be read as a marker of pharmacological response rather than as an ergogenic or adverse effect, and it should not be taken as evidence that caffeine reliably lowers heart rate in this population [49,50]. Blood lactate did not differ significantly between conditions (g = 0.30) but was estimated with very wide uncertainty, and blood glucose was essentially unchanged (g = 0.00); the non-significant lactate result should not be read as evidence of no metabolic effect, given the few contributing studies, heterogeneous protocols, and variable sampling timing [56]. Several mechanistically relevant variables heart rate variability, hydration and sweating, muscle-damage and immune markers, and oxidative stress could not be pooled, an important gap because thermoregulatory, hydration, and recovery considerations may interact with menstrual-cycle phase and hormonal status [57]. Because this domain aggregates indicators of different biological meaning, its pooled value is descriptive only and was rated very low certainty.

Perceptual responses showed the largest domain-level effect (g = 0.42) and are the most mechanistically interpretable result in the review. The effect was driven by ratings of perceived exertion, which were reliably lower after caffeine (g = 0.35, positive values coded as reduced RPE); by antagonizing adenosine receptors, caffeine attenuates central fatigue signalling and the subjective perception of effort and pain, so athletes feel they are exerting less under the same external load [56]. This provides a parsimonious link to the physical and sport-specific running benefits: if a workload feels easier, athletes may sustain higher outputs for longer under accumulating fatigue, particularly late in a match [50,53]. Related constructs felt arousal, pleasure, perceived pain, and perceived fatigue could not be pooled despite their theoretical centrality and warrant systematic measurement [56]. An important caveat governs this domain: perceived exertion is subjective, and caffeine’s perceptible effects, including increased alertness, diuresis, tremor, or palpitations, can compromise blinding integrity and bias subjective ratings in the expected direction [13,14]. Partial unblinding may also influence effort-dependent physical or sport-specific performance outcomes, because expectancy can plausibly alter motivation, pacing, or willingness to sustain maximal or near-maximal effort during testing. Accordingly, the perceptual domain was rated moderate certainty after a one-level downgrade for risk of bias, specifically the risk of compromised blinding caused by caffeine’s recognizable effects; this judgment does not treat subjectivity alone as the reason for downgrading.

### 4.4. Cognitive Performance

Cognitive performance was the least developed domain and supports no firm conclusion. The pooled estimate was small and favorable but non-significant (g = 0.37, *p* = 0.083), and the inferential basis was extremely limited, with only four studies and robust-inference degrees of freedom of approximately 2.6. Neither reaction time (g = 0.37) nor cognitive accuracy (g = 0.42) reached significance, and both carried uninformatively wide confidence intervals. The direction is at least consistent with caffeine’s established effects on alertness, vigilance, and attention, plausibly relevant to the perceptual and decision-making demands of team sports [50,58], but the data are far too sparse to distinguish a genuine effect from sampling noise [59]. This domain was rated very low certainty, downgraded for both imprecision and inconsistency; the implication is not that caffeine fails to affect cognition, but that the question has barely been studied with sport-relevant cognitive tasks [60].

### 4.5. Moderating Factors

Across the exploratory moderator analyses, no statistically reliable moderation was found by dose, timing of intake, formulation or source, sport type, competitive level, habitual caffeine intake, or blinding status. Neither linear nor quadratic dose–response meta-regressions provided evidence of a reliable dose–response pattern. Although current position statements commonly discuss caffeine within the range of approximately 3–6 mg·kg^−1^ and caution against unnecessary higher doses because of side effects, the present dataset does not identify an optimal dose or timing strategy for female team-sport athletes [10,59]. Descriptively, the physical-performance estimate was numerically lower in studies classified as habitually high caffeine consumers than in those classified as low consumers (g = 0.11 vs. 0.34), but the between-group moderation test was not statistically significant (*p* = 0.096). Therefore, this pattern should not be interpreted as evidence that habitual caffeine intake attenuates the acute ergogenic response. Rather, it should be viewed as a hypothesis-generating observation in the context of mixed prior evidence, including well-controlled work finding no significant moderation and tolerance studies suggesting that regular intake may influence caffeine responsiveness [12,61].

Beyond the formal moderator results, menstrual-cycle phase, hormonal-contraceptive use, and habitual caffeine intake remain important variables for interpreting caffeine responses in female athletes. These factors may influence caffeine pharmacokinetics, perceived exertion, tolerance, sleep, and the likelihood of ergogenic or adverse responses. However, menstrual-cycle phase and hormonal-contraceptive use were reported or controlled inconsistently across the included trials and therefore could not be modelled with confidence. Habitual caffeine intake was reported more frequently, but the reporting format varied across studies; therefore, it was coded descriptively and interpreted only as an exploratory study-level moderator. Accordingly, the absence of reliable moderation should be interpreted as a limitation of the current evidence base and reporting quality, not as evidence that these variables are biologically irrelevant. Importantly, the absence of statistically significant moderation should not be taken as proof that factors such as caffeine dose, timing of intake, sport type, or habitual caffeine intake are unimportant, or that clinically or practically relevant modifiers are absent: with uneven numbers of studies across moderator levels, few clusters at several levels, and a median per-study power of only about 8–9% for a small effect, the present dataset was very likely underpowered to detect genuine moderating effects, which may therefore have gone undetected rather than being truly absent.

### 4.6. Practical Implications and Future Research

Because the included evidence mainly reflects young adult or adult female team-sport athletes, these practical implications should be applied primarily to comparable populations and should not be generalized to adolescent athletes or other developmental age groups without further evidence. Current evidence does not justify a blanket claim that acute caffeine improves competitive performance in female team-sport athletes; any benefit is outcome-specific, context-dependent, and modest. In the trials reviewed here, caffeine was most commonly administered at approximately 3–6 mg·kg^−1^ around 60 min before exercise; at these commonly studied doses, caffeine may provide small, context-dependent improvements in selected explosive and repeated high-intensity physical outputs measured in controlled tests, such as jumping, sprinting, change in direction, repeated-sprint ability, and high-intensity running, alongside a reduction in perceived exertion that may help sustain output under fatigue [10,11,15]. Practitioners should not expect caffeine to improve shooting accuracy, decision-making, or other fine technical and tactical skills [62]. Because no dose, timing, or formulation moderator was supported, the evidence does not justify a female team-sport-specific optimal protocol; this should be interpreted as insufficient evidence to identify an optimal dose or timing strategy, rather than as evidence that dose or timing is unimportant. Caffeine should therefore be treated as an individualized acute strategy, trialed in training rather than first attempted in competition, using a dose and timing the athlete has already tolerated, since higher doses have not shown clear evidence of additional benefit and may increase adverse effects [10,63]. Decisions should be weighed against habitual intake, prior individual response, gastrointestinal symptoms, anxiety, sleep disruption, and task demands, and interpreted against the smallest meaningful change in each competitive context rather than statistical significance alone [10,64].

**Figure 6:**
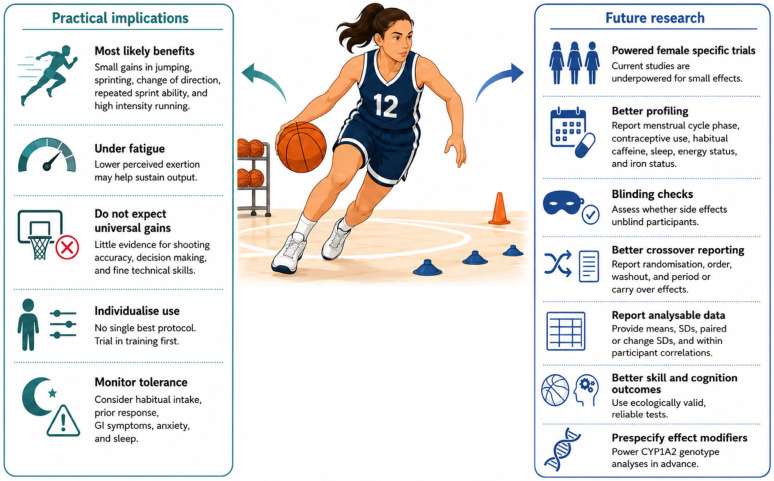
Current evidence-based practices and future directions for acute caffeine use in female team-sport athletes.

Future research should move beyond asking whether caffeine is ergogenic and instead define for whom, under what conditions, and for which outcomes it helps. Adequately powered, female-specific trials are needed; the median per-study power of ≈ 8–9% for a small effect is a structural weakness of the field. Future trials should prospectively report menstrual-cycle phase, hormonal-contraceptive use, habitual caffeine intake, [13,14,54]. Crossover reporting should include randomisation and allocation procedures, condition order, washout duration, and tests for period and carry-over effects [20]. Studies should report condition-level means and standard deviations, paired or change-score standard deviations, and within-participant correlations, preferably alongside individual participant data, so dependent effects can be modelled rather than approximated [18]. Sport-specific skills and cognition, currently the weakest parts of the evidence base, should be prespecified as primary outcomes and assessed with ecologically valid, reliable tests, and CYP1A2 genotype should be prespecified and powered as an effect-modification analysis rather than added post hoc [58,65,66].Current evidence-based practices for future research are summarized in Figure 6.

### 4.7. Strengths and Limitations

This review has several strengths. The synthesis was restricted to female team-sport athletes or independently extractable female subgroups, preserving female-specific uncertainty rather than diluting it through mixed-sex pooling; five domains were analyzed separately rather than collapsed into a single performance construct; and a three-level correlated-and-hierarchical-effects model with CR2 cluster-robust variance, Satterthwaite [18]. Sensitivity analyses across within-study correlations, together with leave-one-out and influence diagnostics, showed the direction of the main findings to be robust: the all-domain estimate remained approximately 0.23–0.24 across assumed correlations, and the physical-performance estimate remained approximately 0.32.

Several limitations should temper these conclusions without overstating their corrosive effect. First, the limitation is largely structural: none of the 26 trials was at overall low risk of bias, the primary studies were uniformly small, and their power to detect small effects was very low, so the literature is better suited to estimating average effects than to resolving moderators or detecting publication bias. Second, no crossover study reported the within-participant correlation between caffeine and placebo conditions, so primary effect sizes were computed from condition-level means and within-condition standard deviations, which does not fully exploit the paired structure of crossover designs. Although the three-level correlated-and-hierarchical-effects model with CR2 robust variance estimation addressed dependence among multiple effect sizes contributed by the same study, incomplete reporting of paired or change-score information in the primary crossover trials means that the exact standard errors and confidence intervals remain conditional on assumptions about the dependence structure. Sensitivity analyses across a plausible range of working correlations left the main estimates stable, but residual uncertainty should still be considered when interpreting the precision of the pooled effects [18]. Third, in several domains the level-3 variance was estimated at the boundary, attributing most heterogeneity to within-study variation; this should not be read as evidence that between-study differences are absent, and the wide prediction intervals make clear that future effects may vary in magnitude and direction. Fourth, the evidence was unevenly distributed across sports and outcomes, leaving cognition, several physiological markers, and perceptual constructs such as pain and fatigue too sparsely studied to pool, and the exploratory subgroup and meta-regression analyses involved multiple comparisons whose null results should not be over-interpreted [54]. Several female-specific and contextual variables were also reported inconsistently across the included trials: menstrual-cycle phase and hormonal-contraceptive use were verified or controlled in only a minority of studies, and habitual caffeine intake was recorded in heterogeneous formats, so none could be modelled with confidence, and their potential modifying roles remain unresolved. Many effects were also derived from controlled laboratory tasks, such as isolated jumps, sprints, or reaction-time protocols, whose ecological validity for actual match play is limited, so the extent to which these findings transfer to competitive team-sport settings is uncertain. Fifth, caffeine’s perceptible side effects may have compromised blinding integrity, a particular concern for subjective perceptual outcomes [13,14]. Sixth, the search was limited to PubMed/MEDLINE and Web of Science, supplemented by backward citation tracking. Although these sources were selected to cover biomedical, nutrition, exercise-science, and multidisciplinary citation-indexed literature, the search was not exhaustive. Eligible studies indexed only in SPORTDiscus, Scopus, Embase, the Cochrane Library, trial registries, or other regional databases may therefore have been missed. In addition, eligibility at the screening stage was restricted to studies with an available English full text. In practice, however, no report was excluded solely because of language after full-text assessment, so the potential for language bias in this review was limited; the main residual concern is the availability of full texts rather than language per se. Finally, although no statistically significant funnel-plot asymmetry was detected, the small number of studies per domain and the predominance of small trials mean that publication bias was undetected but not excluded. The GRADE ratings, ranging from moderate to very low across domains, indicate the appropriate level of confidence in each conclusion.

## 5. Conclusions

Acute caffeine supplementation may provide small, outcome-specific benefits for selected physical-performance outcomes and reduced perceived exertion in female team-sport athletes. The most consistent evidence concerns explosive and repeated high-intensity physical outputs, whereas the evidence for sport-specific skills and cognitive outcomes remains uncertain, resting on few studies per outcome and not supporting a general benefit; physiological outcomes were direction-aligned for analysis but aggregate indicators of differing clinical desirability and should therefore be interpreted as response patterns rather than as uniform benefit or harm. Exploratory moderator analyses did not provide statistically reliable evidence that effects differed by dose, timing of intake, formulation or source, sport type, competitive level, habitual caffeine intake, or blinding status. Therefore, current evidence does not identify an optimal caffeine protocol or support dose-, timing-, formulation-, habitual-intake-, sport-, or population-stratified recommendations. Future adequately powered female-specific trials should prospectively report menstrual-cycle phase, hormonal-contraceptive use, habitual caffeine intake, adverse symptoms, and blinding integrity to clarify individual and context-specific caffeine responses.

## Figures and Tables

**Table 1:** Inclusion and exclusion criteria based on the PICOS framework.

Element	Inclusion Criteria	Exclusion Criteria
Population (P)	Healthy female team-sport athletes (basketball, volleyball, soccer, handball, rugby, softball, water polo, etc.), with no restriction on age or competitive level	Male or mixed-sex samples from which female data could not be separated; individual-sport athletes; injured or clinical populations
Intervention (I)	Acute (single dose) oral caffeine supplementation, any formulation (capsule/anhydrous caffeine, coffee, energy drink, chewing gum)	Chronic (multi-day, repeated) supplementation; multi-ingredient supplements in which the caffeine effect could not be isolated; non-oral administration
Comparator (C)	Placebo condition, within-participant crossover control	No placebo or no appropriate control condition
Outcomes (O)	At least one of physical performance, sport-specific performance, physiological responses, perceptual responses, or cognitive performance, with reported means and standard deviations or convertible statistics	No extractable quantitative outcome reported; only non-convertible statistics reported
Study design (S)	Randomized, (double-) blind, placebo-controlled crossover design	Non-randomized, uncontrolled, narrative studies, reviews, case reports; conference abstracts used only to trace full texts

**Table 2:** GRADE certainty of evidence by outcome domain.

Outcome Domain	Studies (N)	Risk of Bias	Inconsistency	Indirectness	Imprecision	Publication Bias	Final Certainty
Physical performance	24	Serious (−1): no trial at overall low RoB; predominant judgement “some concerns”	Not serious: moderate heterogeneity and a wide PI, but direction-consistent and stable in leave-one-out/influence analyses	Not serious	Not serious: CI excluded the null	Undetected but not excluded; no additional downgrade	Moderate
Sport-specific performance	13	Serious (−1): no trial at overall low RoB	Serious (−1): high between-study heterogeneity	Not serious	Serious (−1): CI crossed the null	Undetected but not excluded; no additional downgrade	Very low
Physiological responses	12	Serious (−1): no trial at overall low RoB	Serious (−1): high heterogeneity across indicators of diverse biological meaning	Serious (−1): aggregate reflects physiological response patterns rather than a single efficacy outcome	Serious (−1): CI crossed the null	Undetected but not excluded; no additional downgrade	Very low
Perceptual responses	12	Serious (−1): likely compromised blinding due to perceptible caffeine effects	Not serious as the limiting domain	Not serious	Not serious as an additional downgrade, despite PI crossing the null	Undetected but not excluded; no additional downgrade	Moderate
Cognitive performance	4	Serious (−1): no trial at overall low RoB	Serious (−1): high heterogeneity	Not serious	Very serious (−2): only four studies, robust *df* ≈ 2.6, very wide CI/PI	Undetected but not excluded; no additional downgrade	Very low

Note: Certainty started at high because all contributing trials were randomized crossover designs. Because no included trial reached overall low risk of bias, a one-level risk-of-bias downgrade was applied consistently to every outcome domain. “Serious” and “very serious” denote one- and two-level downgrades, respectively; certainty was not rated below very low. CI, confidence interval; PI, prediction interval; RoB, risk of bias.

## Data Availability

The data and analytic materials supporting this review are available on the Open Science Framework (OSF; https://osf.io/6hqfp, 10.17605/OSF.IO/QZYWT, 21 May 2026). These materials include the extracted study-level dataset, the derived effect-size dataset, the outcome-domain and moderator coding, the key analytic assumptions (including the assumed within-study correlation), and the R scripts used for the main, subgroup, and sensitivity analyses.

## Supplementary Materials

The following supporting information can be downloaded at: https://www.mdpi.com/article/10.3390/nu18152429/s1, Table S1: PRISMA 2020 checklist; Table S2: Study characteristics; Table S3: PEDro scale scores; Table S4: Risk-of-bias assessment using RoB 2; Table S5: Main effects pooled by outcome domain; Table S6: Sensitivity to assumed within-study correlation; Table S7: Outcome submetric analyses; Tables S8.1–S8.6: Subgroup analyses; Tables S9.1 and S9.2: Dose, timing, and continuous-moderator meta-regressions; Tables S10.1–S10.3: Publication-bias, leave-one-out, and outlier/influence analyses; Table S11: Conservative sensitivity analysis; Table S12: Full database search strategies.
