# Supplementary material for: Effects of Acute Caffeine Supplementation on Physical, Sport-Specific, Physiological, Perceptual, and Cognitive Outcomes in Female Team-Sport Athletes: A Three-Level Meta-Analysis
Source: Nutrients. 2026 Jul 24;18(15):2429. doi: 10.3390/nu18152429 (PMC13467733; doi:10.3390/nu18152429)
Supplement: Supplementary file 1 [file nutrients-18-02429-s001.zip › nutrients-4386440-supplementary.pdf]

Supplementary Table S1. PRISMA 2020 Checklist

Section and Topic	Item #	Checklist item	Location where item is reported
**TITLE Effects of Acute Caffeine Supplementation on Physical, Sport-Specific, Physiological, Perceptual, and Cognitive in Female Team-Sport Athletes: A Three-Level Meta-Analysis**			Page 1
Title	1	Identify the report as a systematic review.	Page 1
**ABSTRACT**			
Abstract	2	See the PRISMA 2020 for Abstracts checklist.	Page 1-2
**INTRODUCTION**			
Rationale	3	Describe the rationale for the review in the context of existing knowledge.	Page 2-4
Objectives	4	Provide an explicit statement of the objective(s) or question(s) the review addresses.	Page 4
**METHODS**			
Eligibility criteria	5	Specify the inclusion and exclusion criteria for the review and how studies were grouped for the syntheses.	Page 5
Information sources	6	Specify all databases, registers, websites, organisations, reference lists and other sources searched or consulted to identify studies. Specify the date when each source was last searched or consulted.	Page 4-5
Search strategy	7	Present the full search strategies for all databases, registers and websites, including any filters and limits used.	Page 4
Selection process	8	Specify the methods used to decide whether a study met the inclusion criteria of the review, including how many reviewers screened each record and each report retrieved, whether they worked independently, and if applicable, details of automation tools used in the process.	Page 5-6

Data collection process	9	Specify the methods used to collect data from reports, including how many reviewers collected data from each report, whether they worked independently, any processes for obtaining or confirming data from study investigators, and if applicable, details of automation tools used in the process.	Page 6
Data items	10a	List and define all outcomes for which data were sought. Specify whether all results that were compatible with each outcome domain in each study were sought (e.g. for all measures, time points, analyses), and if not, the methods used to decide which results to collect.	Page 5-7
	10b	List and define all other variables for which data were sought (e.g. participant and intervention characteristics, funding sources). Describe any assumptions made about any missing or unclear information.	Page 6
Study risk of bias assessment	11	Specify the methods used to assess risk of bias in the included studies, including details of the tool(s) used, how many reviewers assessed each study and whether they worked independently, and if applicable, details of automation tools used in the process.	Page 6
Effect measures	12	Specify for each outcome the effect measure(s) (e.g. risk ratio, mean difference) used in the synthesis or presentation of results.	Page 7
Synthesis methods	13a	Describe the processes used to decide which studies were eligible for each synthesis (e.g. tabulating the study intervention characteristics and comparing against the planned groups for each synthesis (item #5)).	Page 5-8
	13b	Describe any methods required to prepare the data for presentation or synthesis, such as handling of missing summary statistics, or data conversions.	Page 6-7
	13c	Describe any methods used to tabulate or visually display results of individual studies and syntheses.	Page 9-16
	13d	Describe any methods used to synthesize results and provide a rationale for the choice(s). If meta-analysis was performed, describe the model(s), method(s) to identify the presence and extent of statistical heterogeneity, and software package(s) used.	Page 7-9
	13e	Describe any methods used to explore possible causes of heterogeneity among study results (e.g. subgroup analysis, meta-regression).	Page 8-9
	13f	Describe any sensitivity analyses conducted to assess robustness of the synthesized results.	Page 8-9

Reporting bias assessment	14	Describe any methods used to assess risk of bias due to missing results in a synthesis (arising from reporting biases).	Page 8
Certainty assessment	15	Describe any methods used to assess certainty (or confidence) in the body of evidence for an outcome.	Page 8

**RESULTS**			
Study selection	16a	Describe the results of the search and selection process, from the number of records identified in the search to the number of studies included in the review, ideally using a flow diagram.	Page 9
	16b	Cite studies that might appear to meet the inclusion criteria, but which were excluded, and explain why they were excluded.	Page 9
Study characteristics	17	Cite each included study and present its characteristics.	Page 9-10
Risk of bias in studies	18	Present assessments of risk of bias for each included study.	Page 10
Results of individual studies	19	For all outcomes, present, for each study: (a) summary statistics for each group (where appropriate) and (b) an effect estimate and its precision (e.g. confidence/credible interval), ideally using structured tables or plots.	Page 11-13
Results of syntheses	20a	For each synthesis, briefly summarise the characteristics and risk of bias among contributing studies.	Page 9-13
	20b	Present results of all statistical syntheses conducted. If meta-analysis was done, present for each the summary estimate and its precision (e.g. confidence/credible interval) and measures of statistical heterogeneity. If comparing groups, describe the direction of the effect.	Page 11-15
	20c	Present results of all investigations of possible causes of heterogeneity among study results.	Page 13-15
	20d	Present results of all sensitivity analyses conducted to assess the robustness of the synthesized results.	Page 15-16

Reporting biases	21	Present assessments of risk of bias due to missing results (arising from reporting biases) for each synthesis assessed.	Page 15
Certainty of evidence	22	Present assessments of certainty (or confidence) in the body of evidence for each outcome assessed.	Page 17
**DISCUSSION**			
Discussion	23a	Provide a general interpretation of the results in the context of other evidence.	Page 19-23
	23b	Discuss any limitations of the evidence included in the review.	Page 22-23
	23c	Discuss any limitations of the review processes used.	Page 23
	23d	Discuss implications of the results for practice, policy, and future research.	Page 23-24
**OTHER INFORMATION**			
Registration and protocol	24a	Provide registration information for the review, including register name and registration number, or state that the review was not registered.	Page 4
	24b	Indicate where the review protocol can be accessed, or state that a protocol was not prepared.	Page 4
	24c	Describe and explain any amendments to information provided at registration or in the protocol.	Page 4
Support	25	Describe sources of financial or non-financial support for the review, and the role of the funders or sponsors in the review.	Page 24
Competing interests	26	Declare any competing interests of review authors.	Page 24
Availability of data, code and other materials	27	Report which of the following are publicly available and where they can be found: template data collection forms; data extracted from included studies; data used for all analyses; analytic code; any other materials used in the review.	Page 24

Supplementary Table S2. Study Characteristics

Study	Year	Sport	Competition level	N	Blinding	Caffeine source	Dose (mg/kg)	Habitual intake	Timing (min)	# effects	Domains measured
Daneghian et al.,2012	2012	Basketball	Trained/Sub-elite	26	Double-blind	Capsule/anhydrous	5	Moderate	70	6	Physiol
Del Coso et al.,2013	2013	Rugby	Elite/Professional	16	Double-blind	Energy drink	3	Low	60	13	Phys, Sport-spec, Physiol, Percept
Lara et al.,2014	2014	Soccer	Amateur/Recreational	18	Double-blind	Energy drink	3	Low	60	17	Phys, Sport-spec, Physiol, Percept
Pérez-López et al.,2015	2015	Volleyball	Elite/Professional	13	Double-blind	Energy drink	3	Unknown	60	21	Phys, Sport-spec, Physiol, Percept
Fernández-Campos et al.,2015	2015	Volleyball	Elite/Professional	19	Double-blind	Energy drink	2	Low	30	7	Phys
Mahdavi et al.,2015	2015	Basketball	Trained/Sub-elite	24	Double-blind	Capsule/anhydrous	5	Moderate	70	6	Phys, Physiol
Ali et al.,2016a	2016	Mixed team sports	Elite/Professional	10	Double-blind	Capsule/anhydrous	6	Low	60	10	Phys, Physiol

Ali et al.,2016b	2016	Mixed team sports	Unknown	10	Double-blind	Capsule/anhydrous	6	Low	60	8	Physiol, Percept, Cog
Stojanović et al.,2019	2019	Basketball	Elite/Professional	10	Double-blind	Capsule/anhydrous	3	Moderate	60	13	Phys, Sport-spec, Percept
Muñoz et al.,2020	2020	Handball	Elite/Professional	15	Double-blind	Capsule/anhydrous	3	Low	60	12	Phys, Sport-spec, Physiol, Percept
Karayigit et al.,2020	2020	Mixed team sports	Elite/Professional	17	Double-blind	Coffee	3, 6	Low	60	42	Phys, Physiol, Percept, Cog
Arazi et al.,2021	2021	Water polo	Trained/Sub-elite	16	Double-blind	Energy drink	1.92	Unknown	15	10	Phys, Sport-spec, Physiol
Karayigit et al.,2021	2021	Mixed team sports	Trained/Sub-elite	17	Double-blind	Capsule/anhydrous	6	High	60	6	Phys, Physiol, Percept
Bougrine et al.,2022	2022	Handball	Elite/Professional	14	Double-blind	Capsule/anhydrous	6	Low	60	4	Phys
Filip-Stachnik et al.,2022a	2022	Volleyball	Trained/Sub-elite	12	Double-blind	Chewing gum	6.4	Moderate	15	6	Phys, Sport-spec
Filip-Stachnik et al.,2022b	2022	Basketball	Elite/Professional	9	Double-blind	Chewing gum	2.3	Moderate	15	7	Phys, Sport-spec

Karayigit et al.,2022	2022	Mixed team sports	Elite/Professional	13	Double-blind	Coffee	3, 6	High	60	10	Phys, Physiol, Percept
Bougrine et al.,2023	2023	Handball	Elite/Professional	20	Double-blind	Capsule/anhydrous	6	Low	60	8	Phys
Siquier-Coll et al.,2023	2023	Volleyball	Trained/Sub-elite	8	Double-blind	Capsule/anhydrous	5	Moderate	60	9	Phys
Bougrine et al.,2024	2024	Mixed team sports	Trained/Sub-elite	16	Double-blind	Capsule/anhydrous	3, 6, 9	Low	60	15	Phys, Percept
Shiu et al.,2024	2024	Softball	Trained/Sub-elite	14	Single-blind	Chewing gum	3.29	Low	25	7	Phys, Sport-spec
Nieto-Acevedo et al.,2025	2025	Basketball	Trained/Sub-elite	12	Double-blind	Capsule/anhydrous	3	Moderate	60	8	Phys, Sport-spec, Cog
Bougrine et al.,2025	2025	Handball	Trained/Sub-elite	17	Double-blind	Capsule/anhydrous	6	Low	60	4	Phys
Quan et al.,2025	2025	Basketball	Trained/Sub-elite	10	Single-blind	Capsule/anhydrous	3, 6, 9	Low	60	18	Phys, Sport-spec, Cog
Mor et al.,2025	2025	Soccer	Trained/Sub-elite	13	Double-blind	Capsule/anhydrous	6	High	60	15	Phys, Sport-spec, Percept
Lyons et al.,2025	2025	Softball	Trained/Sub-elite	6	Double-blind	Capsule/anhydrous	6	Unknown	60	28	Phys, Sport-spec, Percept

TOTAL: 26 studies, 375 participants, 310 effect sizes

Supplementary Table S3. Physiotherapy Evidence Database (PEDro)

Study	The first author	Year of Publication	D1	D2	D3	D4	D5	D6	D7	D8	D9	D10	D11	Total
1	Daneghian et al.,2012	2012	Y	1	0	1	1	0	0	1	1	1	1	7
2	Del Coso et al.,2013	2013	Y	1	0	1	1	1	1	1	1	1	1	9
3	Lara et al.,2014	2014	Y	1	0	1	1	1	1	0	0	1	1	7
4	Pérez-López et al.,2015	2015	Y	1	0	1	1	1	1	1	1	1	1	9
5	Fernández-Campos et al.,2015	2015	Y	1	0	1	1	1	0	1	0	1	1	7
6	Mahdavi et al.,2015	2015	Y	1	0	1	1	0	0	1	0	1	1	6
7	Ali et al.,2016a	2016	Y	1	0	1	0	1	0	1	1	1	1	7
8	Ali et al.,2016b	2016	Y	1	0	1	0	1	0	1	1	1	1	7
9	Stojanović et al.,2019	2019	Y	1	0	1	1	1	1	1	1	1	1	9
10	Muñoz et al.,2020	2020	Y	1	0	1	1	1	0	1	1	1	1	8
11	Karayigit et al.,2020	2020	Y	1	0	1	1	1	1	1	1	1	1	9
12	Arazi et al.,2021	2021	Y	1	0	1	1	1	0	1	1	1	1	8
13	Karayigit et al.,2021	2021	Y	1	0	1	1	1	0	1	1	1	1	8

14	Bougrine et al.,2022	2022	Y	1	0	1	1	0	0	1	0	1	1	6
15	Filip-Stachnik et al.,2022a	2022	Y	1	0	1	0	1	1	1	0	1	1	7
16	Filip-Stachnik et al.,2022b	2022	Y	1	0	1	0	0	1	0	0	1	1	5
17	Karayigit et al.,2022	2022	Y	1	0	1	1	1	0	1	1	1	1	8
18	Bougrine et al.,2023	2023	Y	1	0	1	1	1	0	1	1	1	1	8
19	Siquier-Coll et al.,2023	2023	Y	1	1	0	1	1	1	0	1	1	1	8
20	Bougrine et al.,2024	2024	Y	1	0	1	1	1	1	0	0	1	1	7
21	Shiu et al.,2024	2024	Y	1	0	1	1	0	0	1	0	1	1	6
22	Nieto-Acevedo et al., 2025	2025	Y	1	0	1	1	1	1	1	1	1	1	9
23	Bougrine et al.,2025	2025	Y	1	0	1	1	1	1	0	0	1	1	7
24	Quan et al., 2025	2025	Y	1	0	1	1	0	0	1	1	1	1	7
25	Mor et al.,2025	2025	Y	1	0	1	1	1	1	1	1	1	1	9
26	Lyons et al.,2025	2025	Y	0	0	1	1	1	0	1	1	1	1	7

Table note: “Y” or “1” indicates that the study meets the criterion; “N” or “0” indicates that the study does not meet the criterion.

Supplementary Table S4. Risk bias assessment tool RoB2.

Study	D1	D2	D3	D4	D5	Overall
Daneghian et al.,2012	Some concerns	Some concerns	Low	Low	Some concerns	Some concerns
Del Coso et al.,2013	Some concerns	Low	Low	Low	Some concerns	Some concerns
Lara et al.,2014	Some concerns	Low	Some concerns	Low	Some concerns	Some concerns
Pérez-López et al.,2015	Some concerns	Low	Low	Low	Some concerns	Some concerns
Fernández-Campos et al.,2015	Some concerns	Some concerns	Low	Low	Some concerns	Some concerns
Mahdavi et al.,2015	Some concerns	Some concerns	Some concerns	Low	Some concerns	Some concerns
Ali et al.,2016a	Some concerns	Low	Low	Some concerns	Some concerns	Some concerns
Ali et al.,2016b	Some concerns	Low	Low	Low	Some concerns	Some concerns
Stojanović et al.,2019	Some concerns	Low	Low	Low	Some concerns	Some concerns
Muñoz et al.,2020	Some concerns	Low	Low	Low	Some concerns	Some concerns
Karayigit et al.,2020	Some concerns	Some concerns	Low	Some concerns	Some concerns	Some concerns
Arazi et al.,2021	Some concerns	Some concerns	Low	Some concerns	Some concerns	Some concerns

Karayigit et al.,2021	Some concerns	Low	Low	Some concerns	Some concerns	Some concerns
Bougrine et al.,2022	Some concerns	Some concerns	Some concerns	Some concerns	Some concerns	Some concerns
Filip-Stachnik et al.,2022a	Some concerns	Some concerns	Low	Low	Some concerns	Some concerns
Filip-Stachnik et al.,2022b	Some concerns	High	Some concerns	Low	Some concerns	High
Karayigit et al.,2022	Some concerns	Some concerns	Low	Low	Some concerns	Some concerns
Bougrine et al.,2023	Some concerns	Some concerns	Some concerns	Low	Some concerns	Some concerns
Siquier-Coll et al.,2023	Some concerns	Some concerns	Low	Some concerns	Some concerns	Some concerns
Bougrine et al.,2024	Some concerns	Some concerns	Some concerns	Some concerns	Some concerns	Some concerns
Shiu et al.,2024	Low	Some concerns	Low	Low	Some concerns	Some concerns
Nieto-Acevedo et al., 2025	Low	Some concerns	Low	Low	Some concerns	Some concerns
Bougrine et al.,2025	Low	High	Some concerns	Some concerns	Some concerns	High
Quan et al., 2025	Some concerns	High	Low	Some concerns	Some concerns	High
Mor et al.,2025	Some concerns	Some concerns	Low	Some concerns	Some concerns	Some concerns

Lyons et al.,2025	Some concerns	High	Low	Some concerns	Some concerns	Some concerns

Supplementary Table S5. **Main Effects (pooled by domain)**

Analysis	k (effects)	N (studies)	Hedges g	95% CI low	95% CI high	t	df	p	Magnitude	tau^2 (L3)	omega^2 (L2)	I2 total %	I2 level 2 %	I2 level 3 %	Q	df(Q)	p(Q)	PI low	PI high
OVERALL (all domains)	310	26	0.24	0.13	0.35	4.34	24.5	0.0002	small	0	0.2068	59	59	0	631.1	309	0	-0.7	1.18
Physical performance	137	24	0.32	0.22	0.42	6.51	22	0	small	0	0.158	52.2	52.2	0	220.4	136	0	-0.51	1.15
Sport-specific performance	60	13	0.36	0	0.73	2.13	12.9	0.0526	small	0.2559	0.0091	60.2	2.1	58.2	92.1	59	0.0038	-0.81	1.54

Analysis	k (effects)	N (studies)	Hedges g	95% CI low	95% CI high	t	df	p	Magnitude	tau^2 (L3)	omega^2 (L2)	I2 total %	I2 level 2 %	I2 level 3 %	Q	df(Q)	p(Q)	PI low	PI high
Physiological response	54	12	-0.05	-0.31	0.21	-0.43	9.9	0.6777	trivial	0.0119	0.1829	62.8	59	3.8	133.1	53	0	-1.07	0.97
Perceptual response	39	12	0.42	0.16	0.68	3.61	10.7	0.0043	small	0.0055	0.1116	43.1	41.1	2	79	38	0.0001	-0.38	1.22
Cognitive performance	20	4	0.37	-0.1	0.83	2.72	2.6	0.0833	small	0	0.1901	57.7	57.7	0	35.3	19	0.0128	-1.21	1.94

Columns: k = effects; N = studies; g = Hedges g; tau² = between-study (L3) variance; omega² = within-study (L2) variance; I² split into level-2/level-3; Q = Cochran's Q; PI = 95% prediction interval.

Supplementary Table S6. **Sensitivity to within-study correlation ρ**

**Analysis**	**ES [95% CI] at ρ = 0.6 (primary) / 0.5 / 0.7 / 0.9**
Sensitivity to within-study correlation rho (ES [95% CI], p):	
OVERALL	rho=0.6 (PRIMARY): 0.244 [0.13,0.35] p<0.001 | rho=0.5: 0.244 [0.13,0.36] p=0.000 | rho=0.7: 0.236 [0.12,0.35] p=0.000 | rho=0.9: 0.228 [0.11,0.34] p=0.000
Physical performance	rho=0.6 (PRIMARY): 0.322 [0.22,0.42] p<0.001 | rho=0.5: 0.323 [0.22,0.42] p=0.000 | rho=0.7: 0.321 [0.22,0.42] p=0.000 | rho=0.9: 0.319 [0.22,0.42] p=0.000
Sport-specific performance	rho=0.6 (PRIMARY): 0.364 [-0.00,0.73] p=0.053 | rho=0.5: 0.369 [-0.00,0.74] p=0.051 | rho=0.7: 0.359 [-0.01,0.72] p=0.053 | rho=0.9: 0.350 [-0.01,0.71] p=0.054
Physiological response	rho=0.6 (PRIMARY): -0.050 [-0.31,0.21] p=0.678 | rho=0.5: -0.047 [-0.31,0.21] p=0.692 | rho=0.7: -0.052 [-0.31,0.21] p=0.663 | rho=0.9: -0.048 [-0.31,0.21] p=0.694
Perceptual response	rho=0.6 (PRIMARY): 0.418 [0.16,0.68] p=0.004 | rho=0.5: 0.418 [0.15,0.68] p=0.005 | rho=0.7: 0.418 [0.17,0.67] p=0.004 | rho=0.9: 0.417 [0.17,0.66] p=0.003
Cognitive performance	rho=0.6 (PRIMARY): 0.367 [-0.10,0.83] p=0.083 | rho=0.5: 0.367 [-0.10,0.83] p=0.083 | rho=0.7: 0.367 [-0.10,0.83] p=0.084 | rho=0.9: 0.368 [-0.10,0.84] p=0.087

Supplementary Table S7. **Outcome Sub-Metrics (within each domain, pooled where ≥3 studies)**

Specific outcome sub-metrics within each broad domain (pooled where >=3 studies)

Broad domain	Outcome sub-metric	k (effects)	N (studies)	Hedges g	95% CI low	95% CI high	p
Physical performance	Jump	41	17	0.29	0.2	0.38	0
	Anaerobic power	26	9	0.3	0.06	0.55	0.0216
	Muscular strength	20	8	0.31	-0.03	0.65	0.0672
	Sprint / speed	19	6	0.39	0.13	0.64	0.0116
	Agility / change-of-direction	15	11	0.42	0.02	0.82	0.0434
	Repeated-sprint ability	12	3	0.43	0.14	0.72	0.0207
	Muscular endurance	4	2	Descriptive only* (N<3)			
Sport-specific performance	Projectile / ball velocity	22	5	0.19	0.03	0.35	0.0307
	Sport-specific locomotor	12	4	0.46	0.27	0.65	0.0029

Broad domain	Outcome sub-metric	k (effects)	N (studies)	Hedges g	95% CI low	95% CI high	p
	Shooting / scoring accuracy	11	3	0.16	-0.03	0.35	0.0782
	Decision-making accuracy	6	1	Descriptive only* (N<3)			
	Game actions / collisions	5	2	Descriptive only* (N<3)			
	Sport-specific jump	2	1	Descriptive only* (N<3)			
	Swimming performance	2	1	Descriptive only* (N<3)			
Physiological response	Heart rate	15	7	-0.3	-0.58	-0.02	0.041
	Blood lactate	10	4	0.3	-0.77	1.38	0.4798
	Blood glucose	7	3	0	-0.2	0.2	0.9787
	Hydration / sweat	6	2	Descriptive only* (N<3)			

**Broad domain**	**Outcome sub-metric**	**k (effects)**	**N (studies)**	**Hedges g**	**95% CI low**	**95% CI high**	**p**
	Heart-rate variability	6	1	Descriptive only* (N<3)			
	Muscle damage / immune	4	1	Descriptive only* (N<3)			
	Oxidative stress	2	1	Descriptive only* (N<3)			
	Metabolic substrates	2	1	Descriptive only* (N<3)			
	Fatigue index	1	1	Descriptive only* (N<3)			
	Aerobic power	1	1	Descriptive only* (N<3)			
Perceptual response	RPE / perceived exertion	27	11	0.35	0.1	0.6	0.0101
	Felt arousal / feeling	8	2	Descriptive only* (N<3)			

Broad domain	Outcome sub-metric	k (effects)	N (studies)	Hedges g	95% CI low	95% CI high	p
	Perceived fatigue	2	2	Descriptive only* (N<3)			
	Pain perception	2	1	Descriptive only* (N<3)			
Cognitive performance	Reaction time	14	3	0.37	-0.5	1.24	0.2478
	Cognitive accuracy	6	3	0.42	-0.76	1.6	0.3235

Note: * Level-specific estimates based on fewer than three independent studies are presented for descriptive purposes only and should not be interpreted as formal inferential evidence. Formal inferential estimates are reported only when at least three independent studies contributed.

Supplementary Table S8. **Subgroup (Moderator) Analyses**

Supplementary Table S8.1 **Overall — all domains combined**

Moderator	Level	k (effects)	N (studies)	Hedges g	95 % CI low	95 % CI high	p	Magnitude	I2 L2 %	I2 L3 %	Moderation F	q	ddf	Moderation p
domain	Physical performance	137	24	0.32	0.22	0.42	0	small	52.2	0	4.23	4	2.1	0.192
	S port-specific performance	60	13	0.36	0	0.73	0.0526	small	2.1	58.2				
	Physiological response	54	12	-0.05	-0.31	0.21	0.6777	trivial	59	3.8				
	Perceptual response	39	12	0.42	0.16	0.68	0.0043	small	41.1	2				
	Cognitive performance	20	4	0.37	-0.1	0.83	0.0833	small	57.7	0				
dose_cat	Low (<3)	24	3	0.43	-0.36	1.22	0.1765	small	83.9	0	0.23	2	3.2	0.807

Moderator	Level	k (effects)	N (studies)	Hedges g	95% CI low	95% CI high	p	Magnitude	I2 L2 %	I2 L3 %	Moderation F	q	ddf	Moderation p
	Moderate (3-6)	269	22	0.22	0.1	0.35	0.0013	small	54.1	0				
	High (>6)	17	3	0.25	-0.49	0.98	0.3646	small	54.3	2.6				
source	Capsule/anhydrous	170	16	0.23	0.04	0.41	0.0201	small	51.6	1.3	0.99	3	2	0.539
	Coffee	52	2	0.14*	-0.08	0.36	0.0991	trivial	61.9	0				
	Energy drink	68	5	0.33	0.07	0.59	0.0237	small	74.5	0				
	Chewing gum	20	3	0.25	-0.07	0.58	0.0887	small	0	0				
sport	Basketball	58	6	0.18	-0.27	0.63	0.3749	trivial	46.4	17.8	0.37	5	2.1	0.841

Moderator	Level	k (effects)	N (studies)	Hedges g	95 % CI low	95 % CI high	p	Magnitude	I2 L2 %	I2 L3 %	Moderation F	q	ddf	Moderation p
	Volleyball	43	4	0.13	-0.04	0.29	0.0934	trivial	39	0				
	Soccer	32	2	0.19*	0.03	0.34	0.0361	trivial	33.9	0				
	Handball	28	4	0.36	-0.1	0.82	0.0925	small	74.1	0				
	Softball	35	2	0.35*	0.11	0.59	0.0281	small	0	0				
	Rugby	13	1	(insufficient)*										
	Water polo	10	1	(insufficient)*										
	Mixed team sports	91	6	0.22	0.03	0.4	0.0306	small	56.6	0				

Moderator	Level	k (effects)	N (studies)	Hedges g	95 % CI low	95 % CI high	p	Magnitude	I2 L2 %	I2 L3 %	Moderation F	q	ddf	Moderation p
level	Elite/Professional	147	11	0.22	0.13	0.31	0.0003	small	57.8	0	0.01	1	23.6	0.909
	Trained/Sub-elite	138	13	0.24	0.01	0.47	0.0439	small	57.9	5.1				
	Amateur/Recreational	17	1	(insufficient) *										
habitual	Low	165	13	0.26	0.14	0.38	0.0004	small	60.9	0	1.37	2	4	0.352
	Moderate	55	7	0.16	-0.23	0.55	0.3555	trivial	37.2	18.1				
	High	31	3	0.09	-0.05	0.24	0.1298	trivial	28.1	0				
blinding	Double-blind	285	24	0.24	0.12	0.36	0.0004	small	59.4	0	0.11	1	2.2	0.768

Moderator	Level	k (effects)	N (studies)	Hedges g	95 % CI low	95 % CI high	p	Magnitude	I2 L2 %	I2 L3 %	Moderation F	q	ddf	Moderation p
	Single-blind	25	2	0.19*	-0.42	0.81	0.3119	trivial	54.5	0				

Note: * Level-specific estimates based on fewer than three independent studies are presented for descriptive purposes only and should not be interpreted as formal inferential evidence. Formal inferential estimates are reported only when at least three independent studies contributed.

Columns: moderation F / q / ddf / p test whether effect differs across levels of the moderator (HTZ-type robust Wald). '(insufficient)' = single study.

Supplementary Table S8.2 Physical performance

Moderator	Level	k (effects)	N (studies)	Hedges g	95 % CI low	95 % CI high	p	Magnitude	I2 L2 %	I2 L3 %	Moderation F	q	ddf	Moderation p
dose_cat	Low (<3)	19	3	0.29	-0.13	0.71	0.1093	small	78.9	0	0.2	2	3.3	0.825
	Moderate (3-6)	109	20	0.32	0.21	0.44	0	small	42.6	0				

Moderator	Level	k (effects)	N (studies)	Hedges g	95 % CI low	95 % CI high	p	Magnitude	I2 L2 %	I2 L3 %	Moderation F	q	ddf	Moderation p
	High (>6)	9	3	0.51	-0.28	1.29	0.1266	medium	47.6	0				
source	Capsule/anhydrous	95	14	0.32	0.17	0.48	0.0006	small	48.3	0	0.01	3	2.2	0.997
	Coffee	4	2	0.34*	-0.19	0.87	0.1073	small	0	0				
	Energy drink	28	5	0.3	0.06	0.54	0.0261	small	72.3	0				
	Chewing gum	10	3	0.31	-0.09	0.72	0.0917	small	0	0				
level	Elite/Professional	57	11	0.28	0.15	0.41	0.0007	small	47.6	0	0.92	1	21.9	0.349
	Trained/Sub-elite	75	12	0.37	0.19	0.55	0.001	small	57	0				

Moderator	Level	k (effects)	N (studies)	Hedges g	95 % CI low	95 % CI high	p	Magnitude	I2 L2 %	I2 L3 %	Moderation F	q	ddf	Moderation p
	Amateur/Recreational	5	1	(insufficient)*										
habitual	Low	67	12	0.34	0.19	0.5	0.0004	small	55.3	0	4.72	2	3.7	0.096
	Moderate	30	6	0.36	0.08	0.64	0.0219	small	26.4	0				
	High	18	3	0.11	-0.06	0.27	0.1401	trivial	0	0				
sport	Basketball	28	5	0.47	0.29	0.65	0.0016	small	28.3	0	3.73	5	2.4	0.19
	Volleyball	26	4	0.16	-0.12	0.43	0.1726	trivial	0.8	0				
	Soccer	18	2	0.17*	-0.06	0.41	0.0827	trivial	0	0				

Moderator	Level	k (effects)	N (studies)	Hedges g	95 % CI low	95 % CI high	p	Magnitude	I2 L2 %	I2 L3 %	Moderation F	q	ddf	Moderation p
	Handball	20	4	0.41	-0.05	0.88	0.0672	small	78.3	0				
	Softball	10	2	0.22*	-1.27	1.72	0.5359	small	0	10.9				
	Rugby	1	1	(insufficient)*										
	Water polo	7	1	(insufficient)*										
	Mixed team sports	27	5	0.26	0.01	0.5	0.0421	small	35.6	0				

Note: * Level-specific estimates based on fewer than three independent studies are presented for descriptive purposes only and should not be interpreted as formal inferential evidence. Formal inferential estimates are reported only when at least three independent studies contributed.

Supplementary Table S8.3 Sport-specific performance

Main effect: k=60, N=13, g=0.36 [-0.01, 0.73], p=0.054; I²(total)=60.2% (L2=2.1%, L3=58.2%).

Moderator	Level	k	N	Hedges g [95% CI]	p	I²(L2)	Moderation test
**Dose**	Low (<3)	4	2	1.36 (N=2, CI not estimable)*	—	—	not testable
	Moderate (3-6)	50	10	**0.25 [0.08, 0.42]**	**0.009**	7%	
	High (>6)	6	2	-0.16 (N=2, CI not estimable)*	—	—	
**Caffeine source**	Capsule/anhydrous	32	6	0.16 [-0.20, 0.53]	0.305	0%	F=0.73 df=(2, 4.8) p=0.527
	Energy drink	18	4	0.90 [-0.73, 2.53]	0.177	9%	
	Chewing gum	10	3	0.17 [-0.15, 0.49]	0.141	0%	
**Sport**	Basketball	16	4	0.06 [-0.53, 0.64]	0.778	0%	not testable
	Volleyball	12	2	0.27 (N=2, CI not estimable)*	—	—	

Moderator	Level	k	N	Hedges g [95% CI]	p	I²(L2)	Moderation test
	Soccer	5	2	0.30 (N=2, CI not estimable)*	—	—	
	Handball	3	1	insufficient (1 study)*	—	—	
	Softball	18	2	0.24 (N=2, CI not estimable)*	—	—	
	Rugby	4	1	insufficient (1 study)*	—	—	
	Water polo	2	1	insufficient (1 study)*	—	—	
Competition level	Elite/Professional	20	5	**0.37 [0.24, 0.49]**	**0.001**	36%	F=0.06 df=(1, 8.9) p=0.819
	Trained/Sub-elite	36	7	0.42 [-0.45, 1.28]	0.285	0%	
	Amateur/Recreational	4	1	insufficient (1 study)*	—	—	
Habitual intake	Low	21	5	0.26 [-0.12, 0.64]	0.134	0%	F=0.23 df=(1, 5.9)
	Moderate	14	4	**0.19 [0.02, 0.35]**	**0.038**	0%	

Moderator	Level	k	N	Hedges g [95% CI]	p	I²(L2)	Moderation test
	High	1	1	insufficient (1 study)*	—	—	p=0.648
	Unknown (unclassified)	24	3	1.03 [-2.21, 4.28]	0.304	4%	
	Double-blind	50	11	**0.45 [0.04, 0.86]**	**0.036**	5%	
**Blinding**	Single-blind	10	2	-0.07 (N=2, CI not estimable)*	—	—	not testable

Note: * Level-specific estimates based on fewer than three independent studies are presented for descriptive purposes only and should not be interpreted as formal inferential evidence. Formal inferential estimates are reported only when at least three independent studies contributed.

Supplementary Table S8.4 Physiological response

Main effect: k=54, N=12, g=-0.05 [-0.31, 0.21], p=0.678; I^2(total)=62.8% (L2=59.0%, L3=3.8%).

Moderator	Level	k	N	Hedges g [95% CI]	p	I²(L2)	Moderation test
**Dose**	Low (<3)	1	1	insufficient (1 study)*	—	—	not testable
	Moderate (3-6)	53	11	-0.10 [-0.35, 0.15]	0.377	60%	

Moderator	Level	k	N	Hedges g [95% CI]	p	I²(L2)	Moderation test
**Caffeine source**	Capsule/anhydrous	15	6	-0.15 [-0.75, 0.45]	0.523	77%	F=0.13 df=(1, 5.5) p=0.736
	Coffee	26	2	0.06 (N=2, CI not estimable)*	—	—	
	Energy drink	13	4	-0.04 [-0.73, 0.64]	0.831	66%	
**Sport**	Basketball	8	2	-0.16 (N=2, CI not estimable)*	—	—	not testable
	Volleyball	2	1	insufficient (1 study)*	—	—	
	Soccer	5	1	insufficient (1 study)*	—	—	
	Handball	2	1	insufficient (1 study)*	—	—	
	Rugby	5	1	insufficient (1 study)*	—	—	
	Water polo	1	1	insufficient (1 study)*	—	—	
	Mixed team sports	31	5	0.08 [-0.24, 0.41]	0.492	37%	

Moderator	Level	k	N	Hedges g [95% CI]	p	I^2(L2)	Moderation test
Competition level	Elite/Professional	37	6	-0.13 [-0.50, 0.25]	0.413	35%	F=0.32 df=(1, 6.0) p=0.593
	Trained/Sub-elite	11	4	0.04 [-1.12, 1.20]	0.908	65%	
	Amateur/Recreational	5	1	insufficient (1 study)*	—	—	
Habitual intake	Low	35	6	-0.10 [-0.35, 0.16]	0.360	39%	not testable
	Moderate	8	2	-0.16 (N=2, CI not estimable)*	—	—	
	High	8	2	0.20 (N=2, CI not estimable)*	—	—	
	Unknown (unclassified)	3	2	0.21 (N=2, CI not estimable)*	—	—	
Blinding	Double-blind	54	12	-0.05 [-0.31, 0.21]	0.678	59%	not testable

Note: * Level-specific estimates based on fewer than three independent studies are presented for descriptive purposes only and should not be interpreted as formal inferential evidence. Formal inferential estimates are reported only when at least three independent studies contributed.

Supplementary Table S8.5 Perceptual response

Main effect: k=39, N=12, g=0.42 [0.16, 0.68], p=0.005; I²(total)=43.1% (L2=41.1%, L3=2.0%).

Moderator	Level	k	N	Hedges g [95% CI]	p	I²(L2)	Moderation test
**Dose**	Moderate (3-6)	38	12	**0.43 [0.17, 0.68]**	**0.004**	43%	not testable
	High (>6)	1	1	insufficient (1 study)*	—	—	
**Caffeine source**	Capsule/anhydrous	20	7	0.34 [-0.11, 0.78]	0.114	0%	F=2.09 df=(1, 4.4) p=0.216
	Coffee	10	2	0.35 (N=2, CI not estimable)*	—	—	
	Energy drink	9	3	**0.65 [0.16, 1.13]**	**0.029**	49%	
**Sport**	Basketball	2	1	insufficient (1 study)*	—	—	not testable
	Volleyball	3	1	insufficient (1 study)*	—	—	
	Soccer	4	2	0.54 (N=2, CI not estimable)*	—	—	
	Handball	3	1	insufficient (1 study)*	—	—	

Moderator	Level	k	N	Hedges g [95% CI]	p	I²(L2)	Moderation test
	Softball	7	1	insufficient (1 study)*	—	—	
	Rugby	3	1	insufficient (1 study)*	—	—	
	Mixed team sports	17	5	0.40 [-0.26, 1.06]	0.163	39%	
**Competition level**	Elite/Professional	21	6	**0.47 [0.13, 0.82]**	**0.018**	58%	F=3.23 df=(1, 5.0) p=0.133
	Trained/Sub-elite	12	4	0.14 [-0.41, 0.69]	0.452	0%	
	Amateur/Recreational	3	1	insufficient (1 study)*	—	—	
**Habitual intake**	Low	23	6	**0.53 [0.09, 0.96]**	**0.026**	52%	F=1.50 df=(1, 3.1) p=0.306
	Moderate	2	1	insufficient (1 study)*	—	—	
	High	4	3	0.22 [-0.58, 1.03]	0.352	0%	
	Unknown (unclassified)	10	2	0.14 (N=2, CI not estimable)*	—	—	

Moderator	Level	k	N	Hedges g [95% CI]	p	I^2(L2)	Moderation test
**Blinding**	Double-blind	39	12	**0.42 [0.16, 0.68]**	**0.005**	41%	not testable

Note: * Level-specific estimates based on fewer than three independent studies are presented for descriptive purposes only and should not be interpreted as formal inferential evidence. Formal inferential estimates are reported only when at least three independent studies contributed.

Supplementary Table S8.6 Cognitive performance

Main effect: k=20, N=4, g=0.37 [-0.13, 0.87], p=0.093; I^2(total)=57.7% (L2=57.7%, L3=0.0%).

Moderator	Level	k	N	Hedges g [95% CI]	p	I^2(L2)	Moderation test
**Dose**	Moderate (3-6)	19	4	**0.45 [0.12, 0.78]**	**0.026**	53%	not testable
	High (>6)	1	1	insufficient (1 study)*	—	—	
**Caffeine source**	Capsule/anhydrous	8	3	0.32 [-0.89, 1.54]	0.341	53%	not testable
	Coffee	12	1	insufficient (1 study)*	—	—	
**Sport**	Basketball	4	2	0.26 (N=2, CI not estimable)*	—	—	not testable

Moderator	Level	k	N	Hedges g [95% CI]	p	I²(L2)	Moderation test
**Competition level**	Mixed team sports	16	2	0.39 (N=2, CI not estimable)*	—	—	not testable
	Elite/Professional	12	1	insufficient (1 study)*	—	—	
	Trained/Sub-elite	4	2	0.26 (N=2, CI not estimable)*	—	—	
**Habitual intake**	Low	19	3	0.28 [-0.40, 0.97]	0.195	57%	not testable
	Moderate	1	1	insufficient (1 study)*	—	—	
**Blinding**	Double-blind	17	3	0.49 [-0.16, 1.13]	0.078	52%	not testable
	Single-blind	3	1	insufficient (1 study)*	—	—	

Note: * Level-specific estimates based on fewer than three independent studies are presented for descriptive purposes only and should not be interpreted as formal inferential evidence. Formal inferential estimates are reported only when at least three independent studies contributed.

Meta-Regression (continuous moderators)

Supplementary Table S9.1 Dose & timing models

Model	k (effects)	N (studies)	Term	beta	95% CI low	95% CI high	SE	p	df	x range
Dose (mg/kg) -> OVERALL, linear	310	26	intercept	0.2413	0.123	0.359	0.0572	0.0003	24.5	1.92-9.00
			dose_mgkg	0.0229	-0.035	0.08	0.0231	0.3624	5.6	
Dose (mg/kg) -> OVERALL, quadratic	310	26	intercept	0.2476	0.088	0.407	0.076	0.0044	17.7	1.92-9.00
			dose_mgkg	0.0254	-0.037	0.087	0.0256	0.3578	6.2	
			dose_mgkg^2	-0.0021	-0.053	0.049	0.0174	0.9127	3.5	
Dose (mg/kg) -> PHYSICAL, linear	137	24	intercept	0.33	0.217	0.443	0.0544	0	21.6	1.92-9.00
			dose_mgkg	0.0418	-0.052	0.136	0.0349	0.2926	4.3	
Dose (mg/kg) -> PHYSICAL, quadratic	137	24	intercept	0.3193	0.156	0.482	0.0759	0.0009	14	1.92-9.00

Model	k (effects)	N (studies)	Term	beta	95% CI low	95% CI high	SE	p	df	x range
			dose_mgkg	0.0379	-0.044	0.119	0.0368	0.3269	10.5	
			dose_mgkg^2	0.0031	-0.034	0.04	0.0126	0.8198	3.6	
Timing (min) -> OVERALL, linear	310	26	intercept	0.2347	0.116	0.353	0.0575	0.0004	23.6	15.00-70.00
			timing_min	-0.0034	-0.014	0.007	0.0042	0.4456	6.3	
Timing (min) -> PHYSICAL, linear	137	24	intercept	0.322	0.217	0.427	0.0507	0	21.8	15.00-70.00
			timing_min	0.0003	-0.007	0.008	0.0031	0.9231	6.1	

Supplementary Table S9.2. Continuous moderators by outcome

Continuous meta-regressions (slope beta of the moderator; positive = larger ergogenic effect)

Continuous moderator	Outcome	beta (slope)	95% CI low	95% CI high	p	k / N
Caffeine dose (mg/kg)	OVERALL	0.0229	-0.022	0.068	0.3624	310 / 26

Continuous moderator	Outcome	beta (slope)	95% CI low	95% CI high	p	k / N
	Physical performance	0.0418	-0.027	0.11	0.2926	137 / 24
	Sport-specific performance	-0.0339	-0.141	0.073	0.6416	60 / 13
	Physiological response	0.0186	-0.043	0.08	0.6163	54 / 12
	Perceptual response	-0.0109	-0.079	0.057	0.7719	39 / 12
	Cognitive performance	-0.0139	-0.225	0.197	0.9098	20 / 4
Pre-exercise timing (min)	OVERALL	-0.0034	-0.012	0.005	0.4456	310 / 26
	Physical performance	0.0003	-0.006	0.006	0.9231	137 / 24
	Sport-specific performance	-0.0123	-0.04	0.015	0.4166	60 / 13

Continuous moderator	Outcome	beta (slope)	95% CI low	95% CI high	p	k / N
	Physiological response	-0.0223	-0.039	-0.006	0.2091	54 / 12
Other (overall set)	Total sample size	-0.0056			0.6185	310 / 26
	Publication year	0.0156			0.383	310 / 26
	Habitual intake (ordinal)	-0.0754			0.2319	251 / 23

Publication Bias & Sensitivity

Supplementary Table S10.1 Egger regression (RVE)

Analysis	Egger bias	SE	t	p	Note
OVERALL	4.131	2.257	1.83	0.1061	OK (>=10 studies)
Physical performance	0.905	2.183	0.41	0.6874	OK (>=10 studies)
Sport-specific performance	4.692	4.752	0.99	0.4229	OK (>=10 studies)

Analysis	Egger bias	SE	t	p	Note
Physiological response	2.991	3.037	0.98	0.3721	OK (>=10 studies)
Perceptual response	1.603	5.758	0.28	0.8104	OK (>=10 studies)
Cognitive performance	24.502	10.983	2.23	0.2462	Descriptive only* (4 studies)

Note: * Egger regression results based on fewer than 10 independent studies are presented for descriptive purposes only; the corresponding p value should not be interpreted as formal evidence of small-study effects.

Supplementary Table S10.2 Leave-one-out sensitivity

Analysis	Full ES	LOO min	LOO max	Stability
OVERALL	0.24	0.21	0.28	stable
Physical performance	0.32	0.3	0.34	stable
Sport-specific performance	0.36	0.22	0.43	review (few studies)
Physiological response	-0.05	-0.1	0.02	stable

Analysis	Full ES	LOO min	LOO max	Stability
Perceptual response	0.42	0.36	0.47	stable
Cognitive performance	0.37	0.28	0.49	review (few studies)

Supplementary Table S10.3 Outlier / influence diagnostics

Supplementary Table S11. Conservative sensitivity analysis: one effect size per study per outcome domain

Outcome domain	Effect sizes in primary model (k)	Effect sizes after reduction (= studies)	Hedges g	95% CI	p	tau²	I² (%)	95% PI	Primary-model g
Physical performance	137	24	0.291	0.16 to 0.42	<0.001	0.030	0	-0.07 to 0.65	0.32
Sport-specific performance	60	13	0.365	0.03 to 0.70	0.034	0.213	64	-0.60 to 1.33	0.36
Physiological response	54	12	-0.001	-0.24 to 0.24	0.990	0.077	45	-0.61 to 0.61	-0.05
Perceptual response	39	12	0.378	0.18 to 0.57	<0.001	0.020	15	0.05 to 0.71	0.42
Cognitive performance	20	4	0.339	-0.01 to 0.69	0.056	0.024	18	-0.20 to 0.88	0.37

Note. Within each study and outcome domain, the multiple dependent effect sizes contributing to the primary three-level model were combined into a single effect size by variance-weighted aggregation of correlated estimates (compound-symmetric working structure, $\rho = 0.6$). Each study therefore contributes exactly one effect size per domain, and the aggregated data were synthesised with a conventional two-level random-effects model (REML). This analysis removes any possibility that studies contributing many effect sizes exert disproportionate influence on the pooled estimates. Domain-level estimates were materially unchanged relative to the primary model. The only change in inferential status was for sport-specific performance, which was borderline in the primary model ($P = 0.053$) and reached nominal significance here ($P = 0.034$); because this reduction discards the repeated-measurement, multi-dose, and shared-placebo structure that the three-level model is

designed to accommodate, it is reported as a robustness check and the more cautious primary interpretation is retained. g, Hedges' g; CI, confidence interval; PI, prediction interval.

Outliers (|std resid|>3): 5; overall ES with outliers removed = 0.24 [0.13, 0.34] (vs full 0.24)

**Outlier effect**	**g, std. residual z**
Daneghian et al.,2012 — TAC	g=-1.6, z=-3.33
Arazi et al.,2021 — 50 m swimming (s)	g=2.8, z=3.82
Arazi et al.,2021 — 100 m swimming (s)	g=2.4, z=3.35
Arazi et al.,2021 — fatigue index	g=-2.32, z=-4.0
Bougrine et al.,2023 — 5m Shuttles Run Test PD	g=2.17, z=3.21

Supplementary Table S12. Full database-specific search strategies

Source	Platform / database	Date searched	Time span	Filters and limits	Records retrieved	Exact search strategy
Electronic database	PubMed/MEDLINE	30 April 2026	From database inception to 30 April 2026	No publication-year restriction. No language restriction was applied at the search stage. Only studies with an available English full text were eligible at the screening stage.	201	((female[MeSH Terms] OR female*[Title/Abstract] OR women[Title/Abstract] OR woman[Title/Abstract] OR girl*[Title/Abstract]) AND (athlete*[Title/Abstract] OR player*[Title/Abstract] OR "team sport*"[Title/Abstract] OR basketball[Title/Abstract] OR soccer[Title/Abstract] OR football[Title/Abstract] OR volleyball[Title/Abstract] OR handball[Title/Abstract] OR rugby[Title/Abstract] OR hockey[Title/Abstract] OR futsal[Title/Abstract] OR netball[Title/Abstract] OR lacrosse[Title/Abstract] OR "water polo"[Title/Abstract] OR softball[Title/Abstract]) AND (caffeine[MeSH Terms] OR caffeine[Title/Abstract] OR caffeinated[Title/Abstract] OR "1,3,7-

Source	Platform / database	Date searched	Time span	Filters and limits	Records retrieved	Exact search strategy
						trimethylxanthine"[Title/Abstract] OR "caffeine ingestion"[Title/Abstract] OR "caffeine supplementation"[Title/Abstract] OR "caffeine gum"[Title/Abstract] OR coffee[Title/Abstract] OR "energy drink*"[Title/Abstract]) AND (random*[Title/Abstract] OR placebo[Title/Abstract] OR crossover[Title/Abstract] OR "cross-over"[Title/Abstract] OR trial[Title/Abstract] OR intervention[Title/Abstract] OR "double blind"[Title/Abstract] OR "single blind"[Title/Abstract] OR "placebo-controlled"[Title/Abstract]))
Electronic database	Web of Science Core Collection	30 April 2026	From database inception to 30 April 2026	No publication-year restriction. No language restriction was applied at the search stage. Only studies with an available English full text were eligible at the screening stage.	203	TS=((female* OR women OR woman OR girl*) AND (athlete* OR player* OR "team sport*" OR basketball OR soccer OR football OR volleyball OR handball OR rugby OR hockey OR futsal OR netball OR lacrosse OR "water polo" OR softball) AND (caffeine OR caffeinated OR "1,3,7-trimethylxanthine" OR "caffeine ingestion" OR "caffeine supplementation" OR "caffeine gum" OR coffee OR "energy drink*") AND (random* OR placebo OR crossover OR "cross-over" OR trial OR intervention OR "double blind" OR "single blind" OR "placebo-controlled"))

Table note: The search strategy was constructed using four concept blocks: population/sex, sport/participants, caffeine intervention, and randomized placebo-controlled crossover study design. No language restriction was applied during database searching. At the screening stage, studies were eligible only when an English full text was available to allow reliable extraction of methodological details and numerical outcome data.
